# Global translational reprogramming in *Trichophyton mentagrophytes*-infected keratinocytes

**DOI:** 10.1080/21505594.2026.2710548

**Published:** 2026-08-02

**Authors:** Jun Wan, Yuhong Zhang, Yangxu Deng, Ruihuan Zhang, Kui Yang, Xixi Zhu, Jiayu Zheng, Bishibei Li, Jingcan You, Lu Fu, Youqiang Sun, Yinpengxi Chen, Ziyao Xiao, Xinnan Luo, Ziyu Wang, Yang Yu, Chunxiang Zhang, Wudian Xiao

**Affiliations:** a School of Basic Medical Sciences, Basic Medicine Research Innovation Center for Cardiometabolic Diseases, Ministry of Education, Department of Cardiology, The Affiliated Hospital of Southwest Medical University, Key Laboratory of Medical Electrophysiology, Ministry of Education, Institute of Cardiovascular Research, Nucleic Acid Medicine of Luzhou Key Laboratory, Laboratory Animal Center, School of Clinical Medicine, School of Public Health, Department of Anesthesiology, Southwest Medical University, Luzhou, China; b Songjiang Research Institute, Shanghai Jiao Tong University School of Medicine, Shanghai, China; c College of Intelligent Manufacturing, Changchun Sci-Tech University, Changchun, China; d Comprehensive Technical Service Center, Comprehensive Technical Service Center of Dongshan Customs, Fujian, China

**Keywords:** *Trichophyton mentagrophytes*, keratinocytes, Ribo-seq, ORFs, translation efficiency, miRNA-mediated regulation

## Abstract

Dermatophytosis afflicts approximately 25% of the global population, representing a critical public health burden. *Trichophyton mentagrophytes* (*T. mentagrophytes*), a predominant zoonotic dermatophyte, is a significant contributor to disease morbidity. While the transcriptomic response of keratinocytes–the primary barrier of the skin–to *T. mentagrophytes* infection is well characterized, the genome-wide translational regulatory mechanisms remain unknown. Here, we employed parallel ribosome profiling (Ribo-seq) and RNA sequencing (RNA-seq) to dissect the genome-wide transcriptional and translational landscape of human keratinocytes during *T. mentagrophytes* infection. Our analysis revealed extensive but distinct reprogramming at both transcriptional (3,189 differentially expressed genes) and translational (295 differentially translated genes) levels, with only minimal overlap (5.8% of upregulated genes and 0.8% of the downregulated genes), indicating prevalent post-transcriptional control. We further identified and characterized a vast non-canonical translatome, including 17,564 upstream ORFs (uORFs), 188,357 downstream ORFs (dORFs), and 185,704 lncRNA-derived ORFs (lncORFs). Strikingly, both translatable uORFs and dORFs were associated with significantly enhanced translation efficiency (TE) of their host genes, a phenomenon conserved under infection. Moreover, we demonstrated that miRNAs coordinately repress target gene expression at both transcriptional and translational levels. Functional interactions revealed that the presence of translated uORFs or dORFs could significantly attenuate miRNA-mediated TE suppression. This study provides the first comprehensive map of the keratinocyte translatome during fungal infection, establishing uORFs and dORFs as critical dual-layer regulators in the host anti-fungal defense.

## Introduction

Dermatomycosis, a zoonotic disease caused by dermatophytes (a closely related group of keratinophilic hyaline fungi), has been recognized since before the 19th century [1]. A small subset of dermatophytes can cause communicable diseases, which primarily affect mammals. As a primary host, humans commonly develop dermatophytoses from these fungi [2]. In clinical practice, dermatophytoses caused by dermatophyte infections include “tinea” or “ringworm,” encompassing conditions such as tinea manuum (hand fungus), tinea pedis (athlete’s foot), tinea corporis (body ringworm), tinea cruris (jock itch), tinea capitis (scalp ringworm), and tinea unguium (nail fungus) [3]. Dermatophytoses is a form of chronic fungal infections, and its symptoms vary by location. Typically, mild itching occurs alongside intermittent, ring-shaped patches with scaling borders. Severe cases may involve blisters on feet or scalp swelling with pus, posing a major public health challenge. According to statistics, superficial fungal infections affect over 20% to 25% of the global population [4]. In recent years, the incidence and recurrence rate of these infections are also increasing due to factors such as the growing elderly population, the widespread use of broad-spectrum antibiotics, and the increased application of immunosuppressive agents [4–6]. Their rapidly increasing severity threatens lives and strains global public health infrastructure [7]. The occurrence of dermatophytosis and the distribution of pathogens may be influenced by various factors, including country, ethnicity, lifestyle, socioeconomic status, and climatic conditions [4,8–11]. Dermatophytes can be categorized into three main genera (anthropophilic, zoophilic, or geophilic), and the collective number of species within these genera exceeds fifty [12]. Among them, *Trichophyton mentagrophytes* (*T. mentagrophytes*) is a major etiological agent of dermatophytosis [13] and the second most common causative dermatophyte in China [11].

*T. mentagrophytes*, as a filamentous fungus, has garnered significant attention in the fields of mycology and dermatology due to its ability to cause infections of the skin, hair, nails, and other keratin-rich tissues. *T. mentagrophytes* is a zoophilic dermatophyte, and human infections primarily occur via direct or indirect animal exposure. Similar to other dermatophytes, the pathogenic mechanism of *T. mentagrophytes* infection involves a cascade of critical steps: fungal adhesion, budding, penetration, and tissue invasion [14]. Studies have demonstrated that *T. mentagrophytes* invades keratin-rich host structure by secreting subtilisin, fungalysin, and metalloproteases [15–17]. Concurrently, the host mobilizes a range of sophisticated responses to counteract dermatophyte invasion, involving immediate innate immune responses, complex adaptive immune strategies, and so on [18,19]. Through the transcriptomic analysis of *T. mentagrophytes*-infected rabbit models, we identified 292 differentially expressed genes and 64 differentially expressed long non-coding RNAs (lncRNAs) associated with *T. mentagrophytes* infection responses [20,21]. Recent studies have utilized transcriptomic approaches to examine the transcriptomic reprogramming that occurs during host-pathogen interactions [22]. Keratinocytes represent the main cell type of the first line of defense in the skin immune system. These cells play a crucial role in maintaining an intact skin barrier and initiating immune responses upon microbial invasion or environmental insults, such as those caused by physical or chemical agents [23]. Human keratinocytes exhibited significant secretion of IL-8 and TNF-α when challenged with *T. mentagrophytes* infection [24]. Despite advances in transcriptomic profiling of dermatophyte infections [25], the translational level responses of human keratinocytes to *T. mentagrophytes* infection remain poorly understood, particularly regarding translational regulatory mechanisms underlying host-pathogen interactions.

Ribosome profiling (Ribo-seq) has emerged as a high-resolution methodology for genome-wide analysis of translational dynamics in living cells. Through the deep sequencing of ribosome-protected mRNA fragments (RPFs), it enables the determination of the extent of translation for a particular gene [26]. Ribo-seq has emerged as a cornerstone technology for dissecting translational regulation across diverse biological processes, including developmental biology, cancer progression, and host-pathogen interplay-particularly in delineating immune responses, inflammatory cascades, and microbial pathogenesis mechanisms [27–32]. While the transcriptional response of keratinocytes to *T. mentagrophytes* infection has been documented in previous studies [33], the corresponding translational regulation mechanisms remain insufficiently explored. Ribo-seq can be employed to identify a variety of novel translational events. For example, it can reveal the effects of upstream open reading frames (uORFs), downstream open reading frames (dORFs), and other open reading frames (such as LncORFs) that encode small peptides on the expression of major open reading frames (mORFs) [34–36]. The uORFs are prevalent in vertebrate genomes, and studies have demonstrated that these regulatory elements predominantly attenuate the translational abundance of downstream mORFs [37]. In contrast to the well-characterized regulatory roles of uORFs, the functional impact of dORFs on mORFs remains relatively understudied. Studies have demonstrated that translation of dORFs enhances the translational output of their cognate mORFs [34]. In addition, Ribo-seq is also useful for ribosomal pause site analysis and the study of translational reprogramming in the context of pathogen-host interactions [36]. However, to date, no systematic investigations have been conducted to elucidate the regulatory roles of uORFs and dORFs in modulating the translation efficiency of mORFs during keratinocyte-mediated defense responses against *T. mentagrophytes* infection.

Therefore, systematic investigation of translational control mechanisms in *T. mentagrophytes*-infected human keratinocytes is of great significance. It can substantially enhance our comprehension of the molecular basis underlying keratinocytes’ defense responses against *T. mentagrophytes* infection, thereby contributing to a more in-depth understanding of the pathogenesis and host-pathogen interactions in *T. mentagrophytes*-related cutaneous infections. In our preceding research, we conducted an analysis to identify both coding and noncoding RNAs in keratinocytes subjected to *T. mentagrophytes* infection through the implementation of whole-transcriptome analysis [25,33,38,39], the translational landscape remains largely unexplored. We combined RNA sequencing (RNA-seq) and Ribo-seq analyses to systematically profile genome-wide transcriptional and translational dynamics in keratinocytes during *T. mentagrophytes* infection. This dual-dimensional approach reveals discordant regulatory patterns between messenger RNA (mRNA) abundance and ribosome engagement, uncovers translationally prioritized pathways (e.g. DNA repair, glycoconjugate biosynthesis), and identifies *cis*-regulatory elements (uORFs/dORFs) and microRNA (miRNAs) that orchestrate translational efficiency (TE). Collectively, these findings establish a multi-layered framework of keratinocyte adaptation to fungal challenge, extending beyond transcriptional regulation alone.

## Materials and methods

### Cell culture and conidia preparation of the *T.* *mentagrophytes*

The immortalized human keratinocyte cell line HaCaT was obtained from the American Type Culture Collection (ATCC; Manassas, VA, USA). The cells were cultured and maintained in Dulbecco’s Modified Eagle Medium (DMEM; Thermo Fisher Scientific, cat. no. C11995500BT) supplemented with 10% fetal bovine serum (FBS; Gibco, cat. no. FSP500) under standard cell culture conditions: 37°C in a humidified CO_2_ incubator (Eppendorf, model CellXpert C170) with 5% CO_2_. The culture and conidia preparation of the *T. mentagrophytes* standard strain ATCC MYA-4439 were performed as described in our previous study [25]. Briefly, conidia of *T. mentagrophytes* were inoculated onto Intergrated Potato Medium (prepared in 10-cm-diameter Petri dishes; Haibo Biotechnology, cat. no. HB0233-6) and incubated at 28°C for 5 to 7 d in a biochemical incubator (Beijing Zhongxing Weiye Century Instrument Co., Ltd., model SHP-80). After incubation, conidia were harvested by washing the mycelial mats with 3–4 mL of phosphate-buffered saline (PBS; KeyGEN BioTECH, cat. no. KGL2206-500). To isolate the conidia, the PBS suspension was filtered through Cell Strainer (Shanghai WoHong Biotechnology, cat. no. WHB-50/20/10UM-S) with progressively smaller pore diameters (50 um, 20 um, and 10 um). Subsequently, the conidial suspension was enumerated and finally diluted with PBS to a concentration of 1 × 10^6^ spores/mL.

### *T.* *mentagrophytes* infection

The conidial suspension (1 × 10^6^ spores/mL) was mixed with complete cell culture medium (DMEM +10% FBS) at a 1:10 (v/v) ratio to yield a final concentration of 1 × 10^5^ conidia/mL. When the keratinocytes in 10-cm dishes reached approximately 80% confluence, the old culture medium was removed. The prepared conidial suspension (8 mL) was then used to infect the keratinocytes for 24 h (Inf group). Control keratinocytes were cultured under standard conditions (CK group).

### Cell collection and ribosome profiling

After treatment, the samples were manipulated on ice, washed with pre-cooled PBS (4 mL), and the PBS was then discarded. Subsequently, 1 mL of pre-cooled PBS was added to the cells, which were then scraped off and transferred into a 1.5 mL microcentrifuge tubes (Sangon Biotech, Order no. F607620-9001). The sample was centrifuged at 1000 × g for 10 min at 4°C. The supernatant was discarded, and the cell pellet was resuspended in 1 mL of pre-cooled PBS and transferred to a new nuclease-free EP tube. The sample was centrifuged again at 1000 × g for 10 min at 4°C, and the supernatant was discarded. The cell pellet was then snap-frozen in liquid nitrogen and sent to Guangzhou Epibiotek Co., Ltd. for further processing and sequencing analysis using the SURFSeq 5000 platform (GeneMind).

### Ribosome profiling data analysis

To ensure the accuracy of downstream analyses, the raw sequencing data were initially processed to remove adapter sequences and low-quality reads using Cutadapt (v4.6), thereby generating high-quality data for subsequent analysis [40]. The quality of the sequencing data was evaluated using FastQC, which provided detailed information on the quality distribution, base content distribution, and the proportion of duplicate sequences. To obtain clean reads, we used Bowtie (v1.2.3) software to align the reads to Ribosomal RNA (rRNA) and Transfer RNA (tRNA) sequences to remove rRNA and tRNA-related sequences [41]. The retained reads were then aligned to reference genomes (GRCh38/hg38) and transcriptomes using HISAT2 (v2.1.0) and Bowtie software, respectively. The number of ribosome profiling reads within the open reading frames (ORFs) regions of coding genes was quantified using the featureCounts (v1.6.3) software and normalized to Fragments Per Kilobase of transcript per Million mapped reads (FPKM) using the DESeq2 R package [42,43]. The Pearson correlation coefficients between replicates were calculated to evaluate the reliability of the ribosome profiling data.

### Differential analysis of gene expression at transcriptional and translational levels

To identify the significantly differentially expressed genes at the translational level, we performed differential translation analysis between the CK and Inf groups using the DESeq2 R package [43]. Genes with significant differences in translation were identified based on adjusted *p* values and log2FoldChange (log2FC). Genes with a |log2FC| of ≥ 1 (corresponding to a fold change of ≥2) and an adjusted *p* value < 0.05 between the CK and Inf groups were considered differentially expressed at the translational level. Similarly, to identify differentially expressed genes at the transcriptional level, we conducted differential expression analysis between the CK and Inf groups using the DESeq2 R package. Genes with an absolute |log2FC| greater than 1 and a *p* value less than 0.05 were deemed to be significantly differentially expressed. Next, genes were categorized into nine discrete clusters based on their differential expression patterns (|log2FC| >1) following the methodology described by Lin et al. [44]. Genes demonstrating concordant regulation were defined as those whose expression altered in the same direction (up or down) at both levels. In contrast, genes with discordant regulation displayed divergent dynamics, characterized by alterations in opposite directions or changes at only one level. Enrichment analysis of Gene Ontology (GO) and Kyoto Encyclopedia of Genes and Genomes (KEGG) pathways was carried out on the OmicShare platform (https://www.omicshare.com) [45] with the Ensembl_109 Reference version.

### Translation efficiency analysis

The translational efficiency (TE) of each gene was computed as the ratio of its normalized ribosome profiling (Ribo-seq) FPKM to its corresponding normalized RNA-seq FPKM [46]. Specifically, this is calculated as TE = (Ribo-seq FPKM) / (RNA-seq FPKM) per gene. Prior to downstream analysis, we excluded genes if any sample contained non-computable (NA) or all samples had infinite (Inf) values, ensuring a robust set of genes for further investigation. For differential TE analysis, Ribo-seq and RNA-seq count data were separately normalized using DESeq2’s median-of-ratios method [43], and genes exhibiting absolute fold changes greater than 2 and Benjamini-Hochberg-adjusted *p*-values less than 0.05 were considered significantly changed. Furthermore, Pearson correlation analysis was performed in the R environment to evaluate the global relationship between transcriptional output (mRNA abundance) and translational control. To focus on highly expressed genes at the transcript level, genes with transcript-level FPKM > 0 were retained within each group. From this subset, we then selected those with expression levels in the top 25%, defined as FPKM ≥ the 75th percentile (upper quartile) of the positive FPKM distribution, for subsequent Pearson correlation analysis.

### Sequence feature analysis

Representative transcripts for multi-isoform genes were identified from the GENCODE annotation, selecting those tagged as “Basic” (experimentally validated) or “Primary” (longest isoform per gene). A rigorous quality control pipeline was employed to curate the corresponding coding sequences (CDS). This involved the removal of redundant transcripts sharing identical CDS and the exclusion of transcripts containing incomplete CDS structures. The resulting curated, non-redundant set of complete CDS was defined as the representative CDS for each gene. To quantify the inherent thermodynamic propensity for secondary structure formation, the Normalized Minimal Free Energy (NMFE) was calculated for each representative CDS. The Minimal Free Energy (MFE) was predicted using RNAfold and subsequently normalized by the nucleotide length to yield the NMFE.

### Identification and analysis of the ORFs

Translationally active open reading frames (ORFs) were interrogated from ribosome profiling data using RiboTricer [47] software (https://github.com/smithlabcode/ribotricer) with a defined set of start codons (ATG, TTG, CTG, GTG, ATA). The predicted ORFs were categorized by genomic context into upstream ORFs (uORFs) in 5’UTRs, downstream ORFs (dORFs) in 3’UTRs, and long non-coding RNA-derived ORFs (LncORFs). A curated subset of uORFs and dORFs that did not overlap the canonical coding sequence (CDS) was subjected to further analysis. Subcellular localization prediction was performed with Deeploc-2.1 utilizing its high-throughput (Fast) model. The primary localization was ascribed to the category with the maximal predicted probability. When multiple localizations share the highest probability value, they are classified as multiple localizations. Applying the method of Lin et al. [44], translation status was determined by FPKM (derived from ORF read counts), with an FPKM ≥ 1 defining a translated ORF. Comparative analysis of global sequence properties between translated and untranslated ORF cohorts was conducted, with statistical significance assessed using a two-sided Wilcoxon test.

### miRNA target analysis

Experimentally documented miRNAs expressed in keratinocytes were obtained from our prior research [25]. Putative miRNA targets of mORF genes were interrogated by integrating computational predictions from Miranda (v3.3a) and TargetScan (v7.0). Targets identified by both algorithms constituted an initial candidate set, which was subsequently subjected to refinement based on interaction thermodynamic stability. Specifically, miRNA-mRNA pairs with a calculated free energy ≤ −30 kcal/mol were retained to enhance prediction reliability. Two-sided Wilcoxon tests were utilized to evaluate differences in gene expression and TE between target and nontarget genes.

## Results

### Generation and characteristics of Ribo-seq data

**Figure 1.**
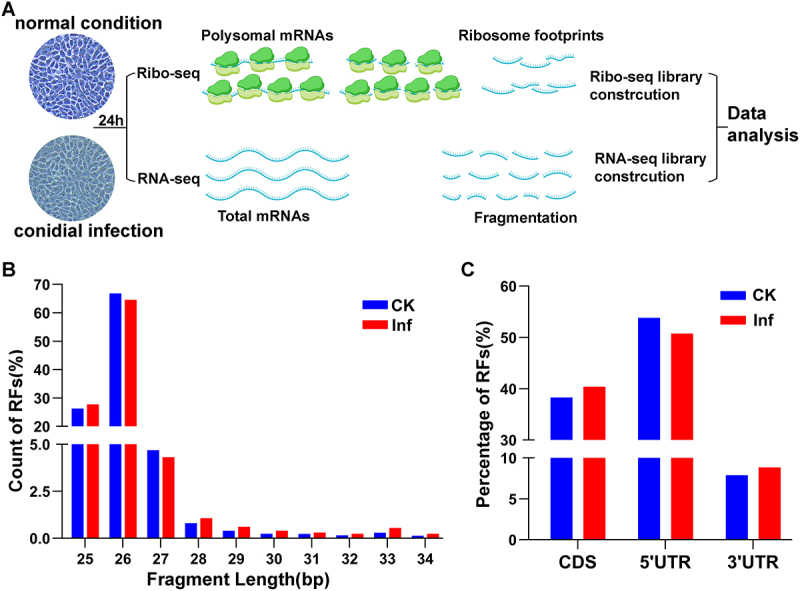
Overview of the experimental design and characteristics of ribosome profiling data analysis. (A) Ribosome profiling, RNA-seq, miRNA-seq, and lncRNA-seq were performed on keratinocytes under normal (CK) and *T. mentagrophytes* infection conditions (Inf). (B) The length distribution of ribosome fragments in the CK and Inf groups. (C) Percentage of ribosome fragment reads located in CDS regions, 5′UTRs, 3′UTRs.

To systematically comprehend the translational response to *T. mentagrophytes* infection at the genome-wide scale, Ribo-seq was performed using next-generation sequencing in both CK group and Inf group (Figure 1(A)). Ribosome profiling of the CK and Inf groups yielded approximately 43.7 million and 40.1 million sequencing reads, respectively (Table S1). Ribosome profiling raw reads were first filtered to remove rRNA and tRNA sequences. Subsequently, the remaining reads from both the CK and Inf groups were subjected to mapping against the human reference genome. An assessment of the Ribo-seq data revealed strong correlation (*R* ≥ 0.98) between biological replicates, supporting the high quality of the data (Figure S1, Table S2).

Studies have demonstrated that ribosome-protected fragments (RFs) typically exhibit a length of approximately 30 nucleotides (nt), although their distribution varies across different organisms [48,49]. The RFs size was determined to be approximately 25 − 26 nt in human keratinocytes in this study (Figure 1(B), Table S3). To compare the RF sizes between the CK and Inf groups and investigate the effects of *T. mentagrophytes* infection on ribosome profiling data, we conducted a comparative analysis. The results demonstrated that the RFs size was largely consistent across the CK and Inf conditions (Figure 1(B)).

Numerous studies have demonstrated the widespread existence of ORFs within untranslated regions (UTRs) across diverse species [50,51]. Our ribosome profiling analysis revealed a unique distribution pattern of ribosome-protected fragments in the CK group, with 38.28% of total reads aligning to coding sequences, while the remaining reads were distributed across non-coding regions: 53.82% in 5′UTRs, and 7.90% in 3′UTRs (Figure 1(C), Table S3). Compared with that in the CK group, the Inf group exhibited distinct ribosome occupancy rates across coding regions and non-coding regions, with 40.40% of reads mapping to coding sequences, 50.75% to 5′UTRs, and 8.85% to 3′UTRs (Figure 1(C)). The increased ribosome reads mapped to 3′UTRs upon *T. mentagrophytes* infection suggested that potential translation events in these regions may play an important role in immune response of human keratinocytes.

### *T.* *mentagrophytes* triggers genome-wide dual-layer reprogramming of gene expression

Previous studies have demonstrated that *T. mentagrophytes* infection alters gene expression at the transcriptional level in human keratinocytes [25,33]. Here, we integrated RNA-seq and Ribo-seq to systematically investigate the effects of *T. mentagrophytes* infection on gene expression regulation at both transcriptional and translational levels in human keratinocytes.

**Figure 2.**
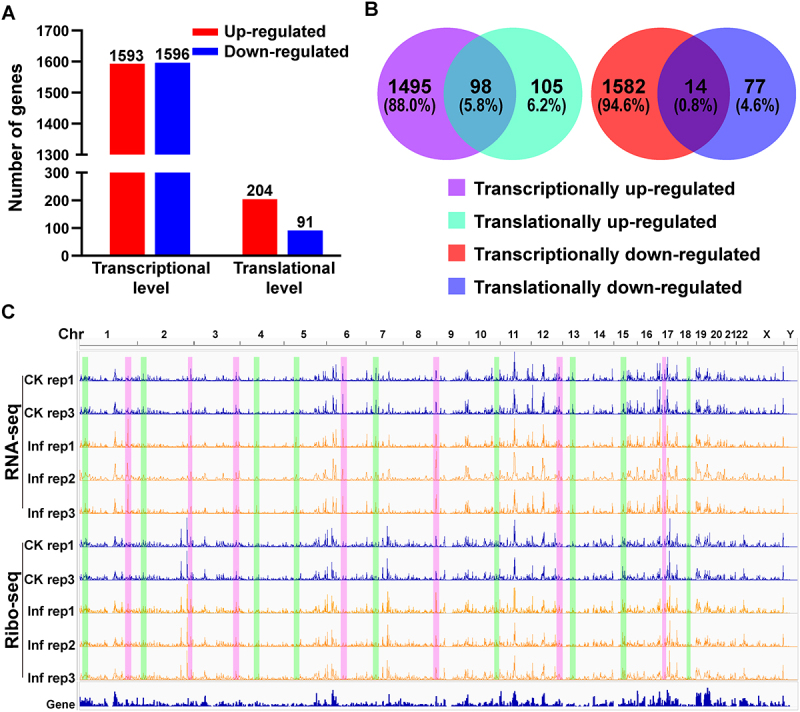
*T. mentagrophytes* infection changes gene expression at the transcriptional and translational levels. (A) Number of genes differentially expressed at the transcriptional or translational level under *T. mentagrophytes* infection. (B) Relationship between responsive genes at the transcriptional and translational levels under *T. mentagrophytes* infection. (C) IGV browser view showing whole-genome RNA-seq and Ribo-seq tracks under *T. mentagrophytes* infection. The light-purple shading indicates that these regions show changes at the two levels under *T. mentagrophytes* infection. The green shading means that these regions show changes at only the transcriptional or translational levels under *T. mentagrophytes* infection.

First, we analyzed the correlation between transcriptional and translational levels using RNA-seq and ribosome profiling data. Results showed that the Pearson correlation coefficients between transcriptional and translational levels were low under both normal and *T. mentagrophytes* infection conditions, with values of 0.22 and 0.48, respectively (Figure S2A, Table S2). Considering that platform-specific differences in sensitivity can lead to technical noise in low-abundance transcripts and thus confound their correlations, we examined transcriptionally highly expressed genes (FPKM > 0 and FPKM ≥ the 75th percentile). Intriguingly, when restricting to highly expressed transcripts, the Pearson correlation coefficient increased from 0.22 to 0.70 in the CK group and from 0.48 to 0.75 in the Inf group (Figure S2A and S2B). These results indicated that technical noise in low-abundance transcripts dampens global correlation, and that translation-transcription concordance is stronger for highly expressed genes. Then, we investigated whether *T. mentagrophytes* infection induced differential gene expression at the two levels. Transcriptomic analysis revealed 1,593 significantly upregulated and 1,596 downregulated genes under *T. mentagrophytes* infection (Figure 2(A), Table S4). In contrast, ribosome profiling identified a distinct translational response, with 204 upregulated and 91 downregulated genes (Figure 2(A), Table S4), suggesting potential post-transcriptional regulation during fungal pathogen adaptation. Interestingly, while the numbers of up- and downregulated genes were comparable at the transcriptional level, a twofold predominance of upregulated over downregulated genes was observed at the translational level. Subsequently, a GO functional enrichment analysis of the differentially expressed genes identified at the transcriptional and translational levels was performed (Figure S3, Table S5). The results revealed an overlap in the enriched biological processes at the two levels, including positive regulation of cell migration, positive regulation of cell motility, regulation of protein serine/threonine kinase activity, response to molecule of bacterial origin, and others.

Notably, a relatively small proportion of the responsive genes exhibited an overlap at the two levels, with 5.8% of the upregulated genes and 0.8% of the downregulated genes being shared (Figure 2(B), Table S4). Analysis of genome-wide RNA-Seq and Ribo-Seq data from all 24 chromosomes (22 autosomes and 2 sex chromosomes) revealed distinct transcriptional and translational dysregulation induced by *T. mentagrophytes* infection (Figure 2(C)). For example, ENSG00000100906, which encodes the NFKB inhibitor alpha, was upregulated at the transcriptional level but exhibited no significant change at the translational level (Figure S4). ENSG00000073331, which encodes the alpha kinase 1, was upregulated only at the translational level (Figure S4).

**Figure 3.**
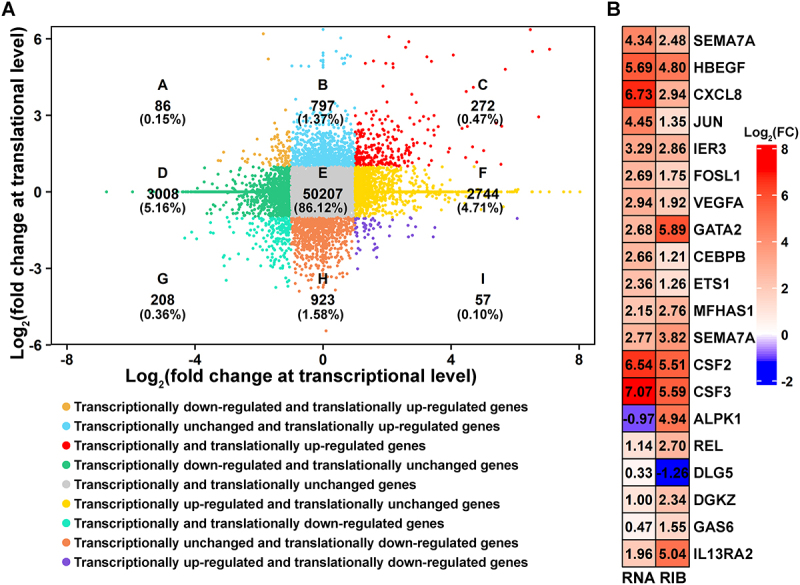
Fold changes of all genes at the transcriptional and translational levels under *T. mentagrophytes* infection. (A) Nine different colors indicate nine different types of responsive genes under *T. mentagrophytes* infection. (B) Fold changes of some important genes related to *T. mentagrophytes* infection at the transcriptional and translational levels. These genes were collected from a previous study [33].

To delineate the genome-wide dynamics of gene expression changes at transcriptional and translational levels, fold changes (FC) were calculated using FPKM. Subsequently, these genes were categorized into nine groups based on the threshold of |log_2_(FC)| >1 (Figure 3(A), Table S6). Comprehensive analysis revealed that 0.83% of differentially expressed genes (272 in Group C and 208 in Group G) exhibited concordant regulation (simultaneous upregulation or downregulation at both transcriptional and translational levels). In addition, the majority of regulated genes (13.07%) displayed discordant expression patterns, distributed across six distinct cohorts: Group A (86 genes), B (797), D (3008), F (2744), H (923), and I (57). Key genes responsive to *T. mentagrophytes* infection in keratinocytes in our prior study showed transcriptional dysregulation [33]. Notably, genes such as *TNFAIP3*, *HBEGF*, *CXCL8*, *CSF2/3*, and *IL13RA2* demonstrated significant upregulation at both transcriptional and translational levels (Figure 3(B), Table S7). In contrast, *ALPK1*, *DLG5*, *DGKZ*, and *GAS6* showed significant upregulation or downregulation exclusively at the translational level.

Sequence features modulate gene expression levels in mammals, a well-established phenomenon across species [52]. To investigate how this regulatory principle operates in *T. mentagrophytes*-infected human keratinocytes, we analyzed the sequence features of concordant genes (C and G groups in Figure 3(A)) and discordant genes (A, B, D, F, H, and I groups in Figure 3(A)) identified in this study. Strikingly, comparative analysis revealed significant differences in sequence features between concordant and discordant genes. Concordant genes exhibited a higher GC content (*p* = 2.3e-05) (Figure S5A, Table S8) alongside a significantly reduced NMFE value (*p* = 4.5-04) within their CDS (Figure S5B, Table S8). Furthermore, to elucidate the functional characteristics and pathway involvement of genes within both concordant and discordant groups, comprehensive GO and KEGG enrichment analyses were conducted (Table S9). Functional enrichment analysis revealed distinct regulatory patterns across transcriptional and translational levels: genes upregulated in the C group at both levels exhibited predominant enrichment in IL-17 signaling pathway (14 genes, *p* = 2.8e-11), TNF signaling pathway (10 genes, *p* = 3.1e-06), and MAPK signaling pathway (14 genes, *p* = 5.3e-05) (Figure S6A). However, genes that were downregulated in the G group at both levels showed significant enrichment in the Herpes simplex virus 1 infection (13 genes, *p* = 9.4e-04), Glycerolipid metabolism (4 genes, *p* = 0.002), and Metabolic pathways (21 genes, *p* = 0.021) (Figure S6B). Functional annotation of discordant genes revealed that the genes in group A (upregulated at the translational level and downregulated at the transcriptional level) demonstrated pronounced enrichment in the Herpes simplex virus 1 infection, Cell cycle, and Peroxisome (Figure S7A). The genes upregulated only at the translational level in group B exhibited significant enrichment in the Metabolic pathways, Mannose type O-glycan biosynthesis, and Hippo signaling pathway (Figure S7B). The genes in group D (downregulated only at the transcriptional level) exhibited significant enrichment in the Herpes simplex virus 1 infection, Peroxisome, Neutrophil extracellular trap formation, and Systemic lupus erythematosus (Figure S7C). The genes upregulated only at the transcriptional level in group F exhibited significant enrichment in the MAPK signaling pathway, Cytokine-cytokine receptor interaction, TNF signaling pathway, and C-type lectin receptor signaling pathway (Figure S7C). The genes downregulated only at the translational level in group H exhibited significant enrichment in the Human T-cell leukemia virus 1 infection and Ribosome biogenesis in eukaryotes (Figure S7D). However, the reliability of enriched pathways in Group I was relatively low (Figure S7E), likely due to insufficient gene representation within this category.

### *T.* *mentagrophytes* infection induces genome-wide translational efficiency reprogramming

**Figure 4.**
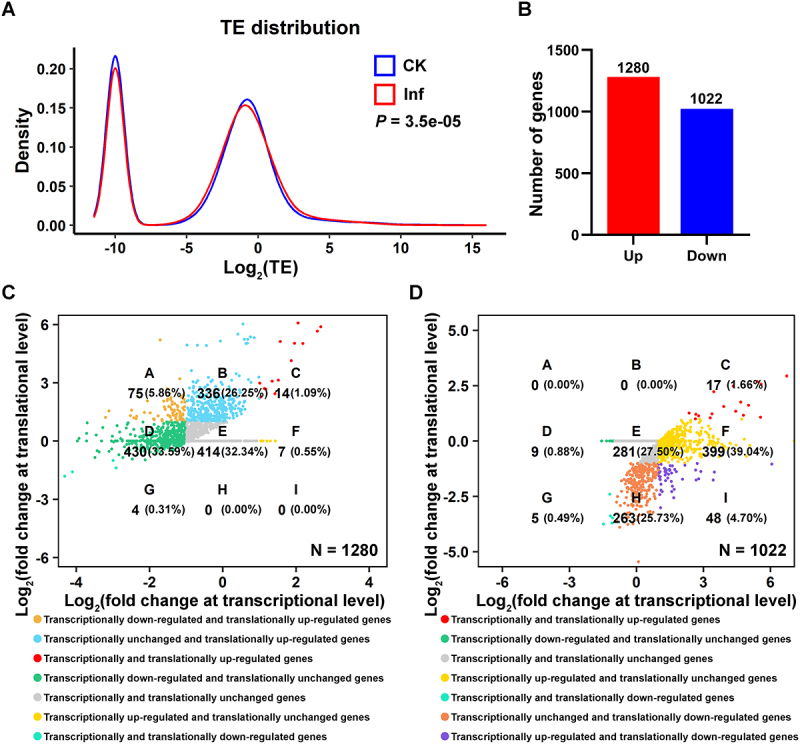
Genome-wide analysis of the translation efficiency (TE). (A) TE analysis between the CK group and Inf group. *p* values were determined by a two-sided Wilcoxon test. (B) Number of genes with significantly changed in TE under *T. mentagrophytes* infection. (C) Distribution of genes with significantly increased TEs in nine different types. (D) Distribution of genes with significantly decreased TEs in nine different types. The nine different types were derived from Figure 3(A).

Translation efficiency (TE), calculated as the ratio of Ribo-Seq to RNA-seq normalized abundances, directly quantifies gene-specific translational regulation [47]. TE values across all genes exhibited significant heterogeneity in the CK group, spanning 8.8 orders of magnitude from 4.0 × 10^−4^ to 2.4 × 10^5^, indicative of complex post-transcriptional regulation mechanisms. Then, we explored whether human keratinocytes can respond to *T. mentagrophytes* infection by examining changes in TEs. Analysis revealed a moderate increase in TEs of overall genes under *T. mentagrophytes* infection (Figure 4(A), Table S10). Notably, transcriptome-wide analysis revealed a modest decrease in global mRNA abundance under infection (Figure S8, Table S10). Furthermore, we found that *T. mentagrophytes* infection significantly altered the TE of 2,302 genes, with 1022 showing decreased TE and 1280 exhibiting increased TE (Figure 4(B); Table S11). These findings suggested that keratinocytes mount a response to *T. mentagrophytes* infection through alterations in the TE of specific genes.

Given that both transcriptional and translational abundance play a role in influencing the final gene TE, we analyzed the distribution of significantly altered TE genes across nine predefined response categories (Figure 3(A)). The analysis demonstrated that differential modulation of transcriptomic/translatomic levels directly drives TE changes. Among genes displaying a marked increase in TE, 59.84% exhibited alterations exclusively at either the transcriptional or translational level (B and D group), 5.86% exhibited alterations at both the transcriptional and translational level (A group) (Figure 4(C)). Among genes displaying a marked reduction in TE, 64.8% exhibited alterations exclusively at either the transcriptional or translational level (Group F exhibited alterations exclusively at the transcriptional level, Group H exhibited alterations exclusively at the translational level), 4.70% exhibited alterations at both the transcriptional and translational level (I group) (Figure 4(D)). In addition, some genes with consistent changes (C group) and no detectable changes (E group) will eventually also manifest changes in gene translation efficiency.

Prior work established that sequence features modulate both transcriptional abundance and translation efficiency [53,54]. To characterize their impact during *T. mentagrophytes* infection, we analyzed sequence features of significantly TE-altered genes in human keratinocytes. Compared to genes with significantly decreased TE, those with significantly increased TE exhibited lower GC content (Figure S9A, Table S12) as well as significantly higher NMFE values in their CDS (Figure S9B, Table S12). Additionally, to further investigate the functional characteristics and associated biological pathways of genes in the significantly TE-elevated and TE-reduced groups, we performed KEGG enrichment analysis. Functional enrichment analysis revealed that genes with increased TE were predominantly enriched in the cell cycle (26 genes, *p* = 5.0 e-07), homologous recombination (11 genes, *p* = 9.5e-06), and p53 signaling pathway (14 genes, *p* = 5.4e-05) (Figure S9C). However, genes with reduced TE were predominantly enriched in the TNF signaling pathway (24 genes, *p* = 5.2 e-09), MAPK signaling pathway (39 genes, *p* = 5.6e-08), autophagy-animal (24 genes, *p* = 4.7e-06), and IL-17 signaling pathway (17 genes, *p* = 5.2e-06) (Figure S9D).

### Genome-wide identification and characterization of ORFs in keratinocytes

**Figure 5.**
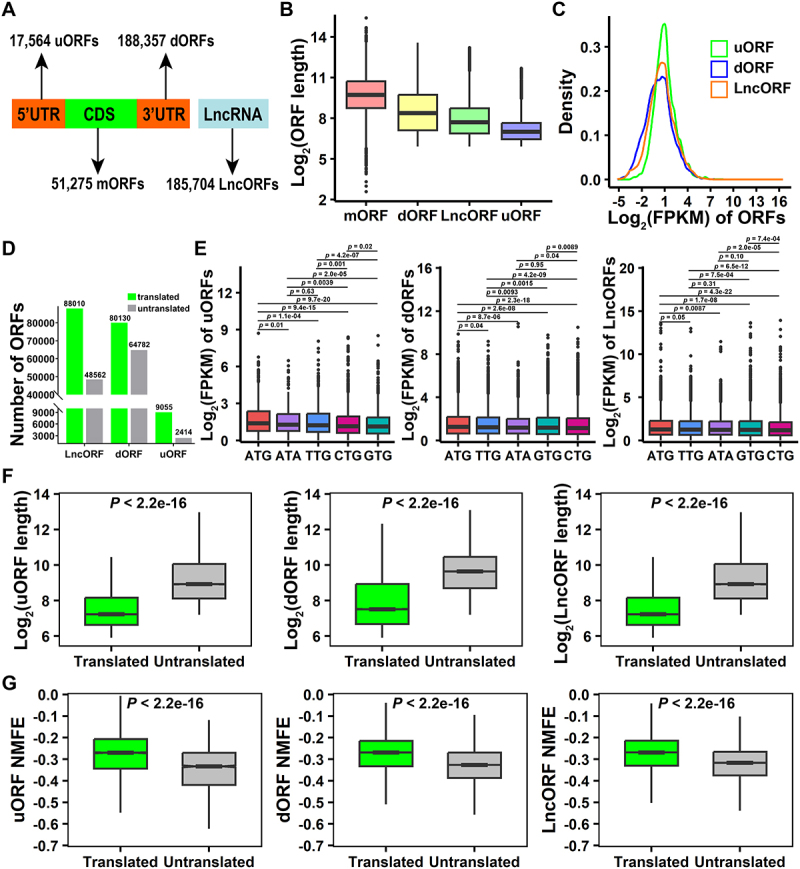
Identification of ORFs. (A) Summary of the ORFs identified, including uORFs, mORFs, dORFs, and LncORFs. (B) Length distribution of ORFs. (C) Expression distribution of uORFs, dORFs, and LncORFs in the CK group. (D) Numbers of translated uORFs, dORFs, and LncORFs. (E) Expression comparison among different start codon usage types for uORFs, dORFs, and LncORFs in the CK group. (F) Comparison of sequence length between translated and untranslated ORFs. (G) Comparison of NMFE between translated and untranslated ORFs. *p* values were calculated using a two-sided Wilcoxon test.

Ribo-seq facilitates transcriptome-wide monitoring of translational activity and enables systematic identification of tissue-specific or condition-dependent ORFs. Using the Ribo-seq data from this study, we performed genome-wide identification of ORFs in keratinocytes by systematically aligning RPFs with the reference genome. Analysis revealed distinct ORF subpopulations across genomic regions: 51,275 canonical mORFs annotated within coding sequences, complemented by 17,564 uORFs in 5’untranslated regions and 188,357 dORFs in 3’ untranslated regions (Figure 5(A), Tables S13-S14). Furthermore, we notably identified 185,704 lncORFs (ORF within long non-coding RNA regions) through integration with our previously characterized lncRNA transcriptome [38] (Figure 5(A), Table S15). We found that mORFs exhibited longer sequence lengths compared to dORFs, uORFs, and lncORFs (Figure 5(B), Table S16), with dORFs, lncORFs, and uORFs showing progressively shorter lengths, respectively. Notably, a low frequency of ATG start codons was observed in dORFs, uORFs, and lncORFs, with 79.3% of dORFs, 80.1% of lncORFs, and 83.9% of uORFs utilizing alternative initiation codons (Figure S10A, Table S16). Subsequently, subcellular localization analysis was performed for these uORFs, dORFs, and lncORFs (Figure S10B, Table S17). Of the 17,564 uORFs analyzed, bioinformatic predictions indicated predominant localization to the cytoplasm (4,112 ORFs), nucleus (5,008 ORFs), mitochondrion (4,743 ORFs), and extracellular (3,384 ORFs). Notably, dORFs and lncORFs exhibited similar subcellular distribution patterns (Figure S10B).

Accumulating evidence has demonstrated that ORFs exert pivotal regulatory roles in diverse biological processes, ranging from the modulation of gene expression, immune regulation, and cellular metabolism to inflammatory responses, across multiple species including humans, mice, and plants [55]. In this study, we initially conducted a comparative analysis of uORFs, dORFs, and lncORFs expression in the CK groups. The expression profiles of dORFs and lncORFs shared common features, indicating a similar mode of regulation. And uORFs demonstrated moderately increased expression levels relative to both dORFs and lncORFs (Figure 5(C), Table S18). Translated ORFs in the CK group were identified using an FPKM threshold ≥ 1, totaling 9,055 uORFs, 80,130 dORFs, and 88,010 lncORFs (Figure 5(D), Table S18). Notably, our analysis of translated ORFs revealed that ATG-initiated ORFs exhibited significantly higher expression than non-ATG-initiated counterparts (Figure 5(E), Table S19). The translated uORFs, dORFs, and lncORFs shared common characteristics, featuring shorter sequence lengths and higher NMFE values (Figure 5(F), G, Table S18).

Then, DESeq2-based differential expression analysis (|log2FC| >1, *p* < 0.05) revealed distinct ORFs regulation patterns between CK and Inf groups (Figure S11, Table S20). The Inf group exhibited subtype-specific ORFs dysregulation, showing 19 downregulated vs 5 upregulated uORFs, 83 downregulated vs 95 upregulated dORFs, and 119 downregulated vs 124 upregulated lncORFs. These data suggested specific roles for ORFs subtypes in keratinocyte defense against *T. mentagrophytes* infection.

### uORFs and dORFs have similar effects on gene translation efficiency regulation

**Figure 6.**
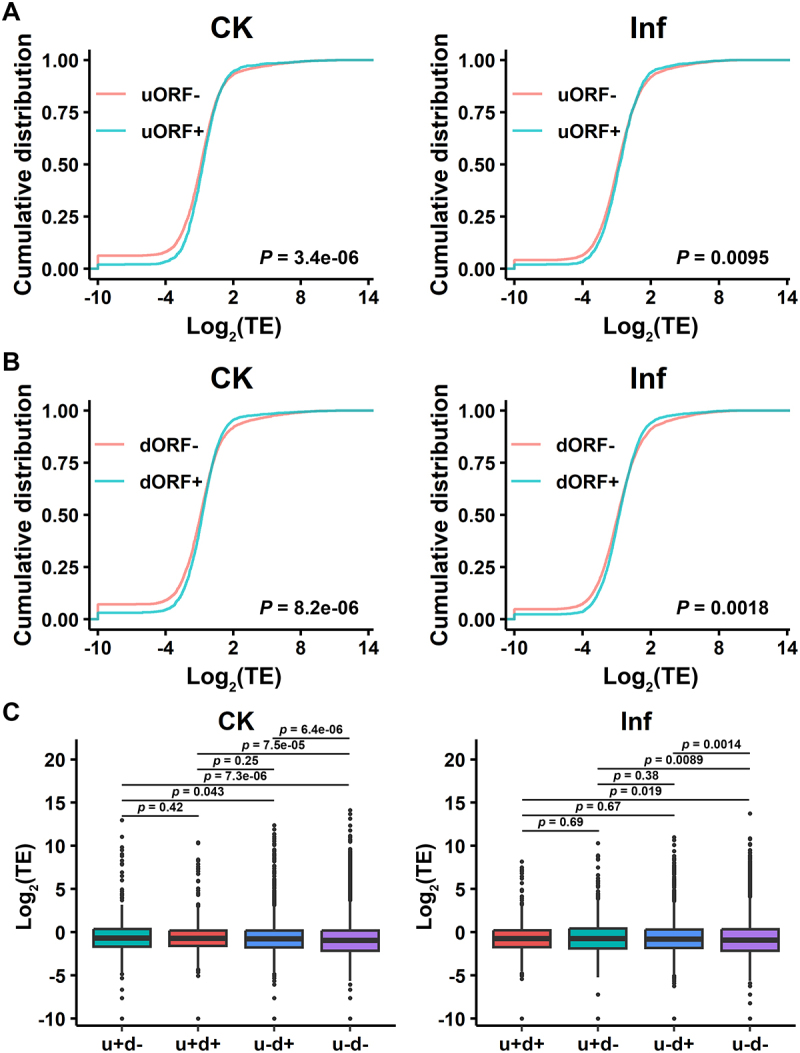
uORFs and dORFs have similar effects on regulating gene TE. (A) Distribution of the TEs of gene with translated uORFs (u+, *n* = 1563) and with untranslated uORFs (u−, *n* = 9137) in the CK group and Inf group. (B) Distribution of the TEs of gene with translated dORFs (d+, *n* = 3925) and with untranslated dORFs (d−, *n* = 6775) in the CK group and Inf group. (C) Comparison of the TEs of gene with translated uORFs and dORFs in the CK and Inf group. Divided into four groups: u+d+ (*n* = 819, presence of translated uORFs and dORFs), u+d- (*n* = 744, presence of translated uORFs and absence of translated dORFs), u−d+ (*n* = 3106, absence of translated uORFs and presence of translated dORFs), and d−u+ (*n* = 6031, absence of translated uORFs and dORFs). *p* values were determined by a two-sided Wilcoxon test.

While uORFs/dORFs modulate mORF translation efficiency (TE) across mammals [34,35,56], their regulatory functions during *T. mentagrophytes* infection of human keratinocytes remain unexplored. We initially examined how translatable uORFs coordinate gene expression at the two levels (transcriptional and translational). Under physiological conditions (CK group), genes harboring translatable uORFs (uORF+) demonstrated elevated expression abundance at both the transcriptional and translational levels compared to genes without translatable uORFs (uORF-) (Figure S12A, Table S21). Under *T. mentagrophytes* infection conditions, both genes harboring translatable uORFs and those without translatable uORFs demonstrated expression abundance similar to that observed in the CK group at both levels (Figure S12B, Table S21). Notably, under both normal and *T. mentagrophytes* infection conditions, genes containing translatable uORFs demonstrated a significantly elevated translational efficiency (TE) compared to genes lacking such translatable uORFs (Figure 6(A), Table S21). Here, we prioritized the candidate gene *BMP4* (a key inflammatory gene) [57] for future functional validation. In the infected group we identified two upstream translated uORFs in the 5′UTR of *BMP4*. Translation of these uORFs was increased, and notably, the TE of the *BMP4* mORF was elevated despite unchanged transcript levels. This pattern suggested a uORF-mediated regulatory mechanism that enhances mORF translation during antifungal infection. Together, these findings provide correlative functional evidence supporting a role for these uORFs in host defense. Furthermore, we examined the association between the increase in translated uORFs and TE. The analysis revealed that increasing the number of uORFs did not enhance TE under either normal conditions or *T. mentagrophytes* infection (single vs multiple uORFs: *p* > 0.99; Figure S12C, Table S21).

In contrast to uORFs, dORFs have been relatively understudied in mammalian systems. Genes were stratified into translatable dORFs (dORF+) and non-translatable dORFs (dORF-) cohorts to evaluate dORF-mediated transcriptional/translational regulation. Our analysis demonstrated that, under normal and *T. mentagrophytes* infection conditions, dORF+ genes exhibited significantly higher expression abundance than dORF- genes, at the two levels (Figure S13A, B, Table S21). Additionally, our analysis revealed a significantly higher TE in genes with translatable dORFs compared to those without translatable dORFs, under both normal and *T. mentagrophytes* infection conditions (Figure 6(B), Table S21). Notably, our analysis also confirmed that an increased number of dORFs enhanced TE. Furthermore, this positive effect of dORFs on gene TE was more pronounced in the CK group (single vs multiple dORFs: *p* = 0.0015; Figure S13C, Table S21).

To gain deeper insights into the combined impact of uORFs and dORFs on gene TE, we categorized genes of mORFs into four distinct groups according to the presence or absence of translatable uORFs and translatable dORFs. Experimental analysis of the CK cohort revealed a descending hierarchy of TE values among mORF groups: the “u+d-” group (presence of uORF, absence of dORF) exhibited the highest TE, followed by the “u+d+” group (presence of both uORF and dORF), then the “u-d+” group (absence of uORF, presence of dORF), while the “u-d-” group (absence of both uORF and dORF) showed the lowest TE (Figure 6(C), Table S21). Notably, under *T. mentagrophytes* infection conditions, mORFs in the “u+d+” group exhibited the highest TE, followed by the “u+d-” group (Figure 6(C), Table S21). These results suggest that dORFs and uORFs exert similar effects on the TE of regulator genes. Under normal physiological conditions, no significant differences in TE were observed between the “u+d+” and “u+d-” groups (*p* = 0.42) or between the “u+d+” and “u-d+” groups (*p* = 0.25). Under *T. mentagrophytes* infection conditions, similar results were obtained. The above findings indicated that both translatable uORFs and dORFs regulate gene TE, though their relative regulatory dominance remains unclear.

### Regulation of gene translation by miRNAs, uORFs, and dORFs

**Figure 7.**
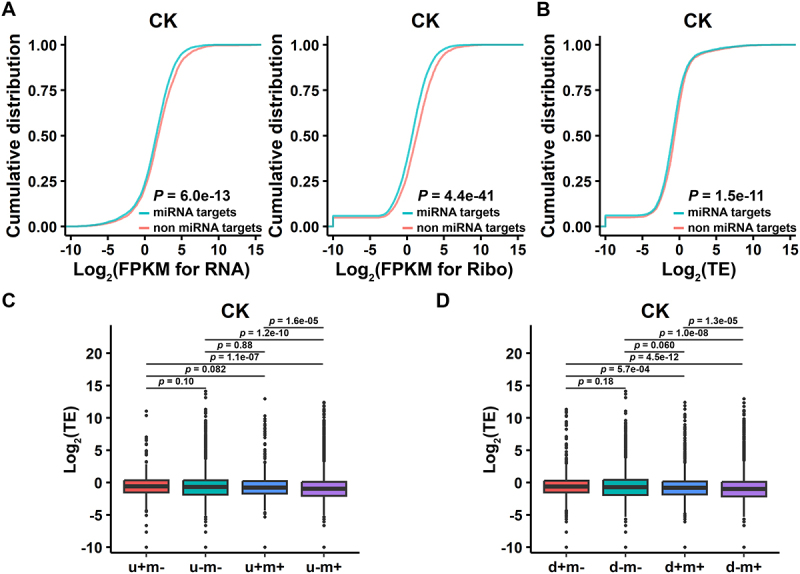
Regulation of gene expression and TE by miRNAs, uORFS, and dORFS under normal conditions. (A) Distribution of gene expression at the transcriptional and translational levels for miRNA targets and non-miRNA targets. (B) Distribution of TEs for miRNA targets and non-miRNA targets. (C) Comparison of TEs across four mRNA categories: “u+m-” (*n* = 587), “u-m-” (*n* = 3065), “u+m+” (*n* = 976), and “u-m+” (*n* = 6072). “u+/u−” denotes mRNAs with or without translated uORFs, and “m+/m−” indicates whether mRNAs are targets of miRNAs or not. (D) comparison of TEs across four mRNA categories: “d+m-” (*n* = 1365), “d-m-” (*n* = 2287), “d+m+” (*n* = 2560), and “d−m+” (*n* = 4488). “d+/d−” denotes mRNAs with or without translated dORFs, and “m+/m−” indicates whether mRNAs are targets of miRNAs or not. *p* values were determined by a two-sided Wilcoxon test.

Animal microRNAs (miRNAs) canonically regulate targets via 3’ untranslated regions (3’UTRs) binding with imperfect complementarity, resulting in transcript repression [58,59]. In our previous study, we identified 2137 expressed miRNAs in 6 small RNA-seq libraries [25]. To investigate their impact on gene expression at both transcriptional and translational levels in keratinocytes, we first identified miRNA target genes, yielding 7653 candidates (Table S22) that contain mORFs. These targets showed significant transcriptional and translational downregulation both basally and during *T. mentagrophytes* infection (Figure 7(A) and S14A, Table S21). Notably, miRNA target genes showed reduced TE both basally and during *T. mentagrophytes* infection, with enhanced suppression under infection (Figures 7(B) and S14B, Table S21). These findings indicated that miRNAs concurrently repress the transcription and translation of mORF‑containing target genes in keratinocytes, thereby modulating host‑response pathways. Among these expressed miRNAs, 209 were differentially expressed (Table S23). To further investigate the impact of these differentially expressed miRNAs on the TE of their target genes, we performed an integrative miRNA-mRNA TE analysis. Specifically, our results demonstrated that, compared with the targets of significantly downregulated miRNAs, the targets of significantly upregulated miRNAs exhibited reduced TE activity during *T. mentagrophytes* infection (Figure S15). This further demonstrated miRNA-mediated TE repression during infection.

To assess coordinated regulation of TE by uORFs, dORFs, and miRNAs, we stratified genes based on combinatorial status: uORF/dORF presence (+) or absence (-), and miRNA targeting. uORF-miRNA interactions generated distinct TE gradients. In both CK and Inf cohorts, mORF groups showed a hierarchical TE decrease: genes with uORFs but not targeted by miRNAs (u+m-) >genes lacking uORFs and not targeted by miRNAs (u-m-) >genes with uORFs and identified as miRNA targets (u+m+) >genes lacking uORFs but identified as miRNA targets (u-m+) (Figure 7(C) and S14C, Table S21). Furthermore, under normal conditions, our analysis revealed that the presence of translated uORFs did not significantly alter TE between miRNA-targeted (u+m+) and non-targeted (u+m-) genes (*p* = 0.082). Conversely, a significant difference (*p* = 1.6e-05) in TE was found between miRNA target genes containing translated uORFs (u+m+) and those lacking translated uORFs (u-m+). The results may suggest that, under basal conditions, translated uORFs emerged as dominant regulators of mORF translation efficiency, significantly attenuating miRNA-mediated suppression. Notably, under *T. mentagrophytes* infection, a significant difference in TE were observed between the “u+m+” and “u+m-” groups (*p* = 2.7e-05) or between the “u+m+” and “u-m+” groups (*p* = 0.009). These findings indicated that miRNAs and uORFs might convergently regulate gene TE during infection, establishing cooperative control where their functional hierarchy requires further resolution.

In terms of dORFs and miRNAs, the TE hierarchy among dORF groups mirrored that of uORF groups: d+m- >d-m- >d+m+ >d-m+ (Figures 7(D) and S14D, Table S21), recapitulating the identical pattern observed in uORF-miRNA interactions. We found that there were no significant differences in TE only between “d+m-” and “d-m-” groups under both normal and *T. mentagrophytes* infection conditions (*p* = 0.18 and *p* = 0.75, respectively). While these results suggested that miRNAs and dORFs may also have a convergent role in regulating gene expression, their functional hierarchy in controlling TE remains to be elucidated.

## Discussion

*T. mentagrophytes* are pathogenic dermatophytes capable of inducing various superficial fungal infections in humans and animals, typically restricted to colonization of the stratum corneum (the outermost epidermal layer) without deeper tissue invasion [60,61]. Although rarely life-threatening, *T. mentagrophytes* infections exhibit high and rising prevalence rates, particularly among immunocompromised individuals, imposing a significant clinical and public health burden [6,62]. Recent studies have employed transcriptomic approaches to investigate gene expression dynamics in dermatophytes. Examples include comparative analyses of the dermatophyte *Arthroderma benhamiae*, which reveal differential protease expression profiles between its host-invading phase and in vitro trophic phase [63]. Additionally, these studies have uncovered transcriptomic reprogramming during host-pathogen interactions [22]. Despite advances in transcriptomic profiling of dermatophyte infections [25], the translational level responses of keratinocytes to *T. mentagrophytes* infection remain poorly understood, particularly regarding translational regulatory mechanisms underlying host-pathogen interactions. Ribo-seq has emerged as a cornerstone methodology for studying gene expression regulation at the translational level, providing genome-wide insights into ribosome occupancy and TE across diverse biological contexts [26]. By combining RNA-seq and Ribo-seq analyses, we delineated divergent transcriptional and translational regulation patterns in *T. mentagrophytes*-infected keratinocytes. This two-level profiling revealed translation-specific control mechanisms that complement transcriptomic responses, offering novel insights into epidermal defense strategies against *T. mentagrophytes* invasion.

### Characteristics of transcriptional and translational regulation in keratinocytes responding to *T.* *mentagrophytes* infection

Transcription produces messenger RNA (mRNA) from DNA templates. Subsequently, translation synthesizes corresponding proteins using mRNA as a template, representing a key step in gene expression [64]. Numerous studies have revealed a prevalent discordance in gene expression regulation between transcriptomic and proteomic levels, with their correlation typically being low [27,65,66]. Hence, conducting in-depth research on the transcription and translation processes is essential for enhancing our understanding of gene expression regulation. Our previous studies have revealed that *T. mentagrophytes* infection significantly impacts gene expression at the transcriptional level [25,38,39]. In this study, we integrated Ribo-seq and RNA-seq data to delineate gene expression changes at transcriptional and translational levels across the entire genome.

Applying a threshold of |log_2_ (FC)| >1, we identified 8,095 genes that were altered at either the transcriptional or the translational level. However, only 480 of these genes showed concordant changes at both levels, whereas 7,615 genes displayed discordant regulation (Figure 3(A)). Notably, the Pearson correlation coefficient between transcriptional and translational levels was below 0.5 under both normal conditions and *T. mentagrophytes* infection, a finding consistent with another study [66]. However, when we restricted the analysis to highly expressed genes (FPKM > 0 and FPKM ≥ the 75th percentile), the correlation increased markedly. We then analyzed the uORF status of key genes responsive to *T. mentagrophytes* infection in keratinocytes, such as *TNFAIP3*, *HBEGF*, *CXCL8*, *CSF2/3*, and *IL13RA2*, which showed significant upregulation at both transcriptional and translational levels. Our results demonstrated that these immune-related genes predominantly fall into the low uORF group (Table S7). This indicates that these immune-related genes (Figure 3(B)) with fewer uORFs tend to show higher correlations in their immune response effect sizes. Further analysis of these altered genes revealed that 3,189 genes exhibited statistically significant changes at the transcriptional level, whereas only 295 genes showed significant alterations at the translational level (Figure 2(A)). Among these significantly changed genes, only 113 were shared between the two levels. This indicates that keratinocytes respond to environmental changes through regulatory mechanisms at both the transcriptional and translational levels [67,68]. However, discordance exists in gene expression regulation between the transcriptomic and translatomic levels, implying a complex, multi-tiered adaptation process. While the functional consequences of the observed translation reprogramming remain to be directly tested, our previous assessment of key cytokine protein levels (including IL-1β, IL-6, TNF-α, and CXCL8) [33] using ELISA provides initial evidence for translational regulation and suggests that translational control may contribute to the keratinocyte’s integrated antifungal response.

Moreover, sequence feature analysis revealed that genes exhibiting concordant changes possessed higher GC content and lower NMFE in their CDS. This distinct sequence signature suggests that mRNAs from concordantly regulated genes are prone to form highly stable, GC-rich secondary structures [69]. Such structural stability is known to influence mRNA metabolism by enhancing transcript stability [70,71] and/or modulating translation efficiency [72]. Furthermore, more stable secondary structures have been shown to slow mRNA turnover, thereby strengthening the correlation between transcript and protein abundance [73]. These findings align with a previous quantitative multi-omics study which observed that the CDS of genes concordantly up-regulated at both transcriptional and translational levels tend to possess higher GC content [74]. Collectively, this sequence-dependent structural stability may mechanistically explain the observed coordinated regulation at both the transcriptional and translational levels for these specific transcripts.

Functional enrichment analysis of genes concordantly upregulated at both levels identified key pathways, such as the IL-17 signaling pathway, TNF signaling pathway, and MAPK signaling pathway, which are significantly associated with keratinocytes’ adaptive responses to *T. mentagrophytes* infection [33]. Notably, genes downregulated at the transcriptional level – irrespective of their translational level changes – demonstrated significant enrichment in the Herpes simplex virus 1 infection pathway (Figure S6B, S7A). The HSV-1 infection pathway encompasses critical innate immune components – including pattern recognition receptors (e.g. TLRs, NLRs), interferon (IFN) signaling cascades, and IFN-stimulated genes (ISGs) – which constitute a first line of defense against diverse pathogens, including fungi [75,76]. In our results, a significant downregulation of TLRs, NLRs, and ISGs was observed at the transcriptional level. This observation suggested that *T. mentagrophytes*, similar to viruses, may actively suppress the transcription of these pathway-associated genes to subvert host antifungal immune defenses, thereby enhancing its infectivity. Research indicated that glycoconjugates, such as the glycocalyx and mucins, shield cells from pathogenic microbial invasion and associated toxin assaults [77]. This protective mechanism preserves plasma membrane integrity and prevents direct interactions between pathogens and cell surface receptors [78,79]. Intriguingly, we observed that genes upregulated solely at the translational level, without corresponding transcriptional changes, were significantly enriched in pathways associated with cell surface glycoconjugate formation. These include Glycosphingolipid biosynthesis-lacto and neolacto series, Metabolic pathway, and Mannose type O-glycan biosynthesis (Figure S7B). This finding suggested that keratinocytes, upon *T. mentagrophytes* infection, may predominantly employ rapid and efficient translational regulation to enhance the synthesis or specific glycosylation of cell surface glycoconjugates. Such a mechanism could potentially serve as a countermeasure to hinder the adhesion and invasion of fungal spores and hyphae, though this requires further experimental validation. In summary, keratinocytes exhibit both interdependent and autonomous gene expression patterns at transcriptional and translational levels during *T. mentagrophytes* infection. This dual-layered regulatory strategy likely enhances cellular adaptability by balancing coordinated responses with pathway-specific adjustments, enabling precise environmental adaptation while maintaining essential biological functions.

### Translational efficiency reprogramming in keratinocytes: a strategic response to *T.* *mentagrophytes* infection

To investigate the impact of *T. mentagrophytes* infection on keratinocyte translation efficiency (TE) and determine whether keratinocytes respond to infection by altering TE for specific genes, we analyzed global gene TEs. This analysis revealed a slight overall increase in gene TEs following infection, with 2,302 genes exhibiting significant TE changes. Given that both transcriptional and translational abundance contribute to a gene’s TE [80], we further examined the distribution patterns of genes with significantly altered TEs. Notably, for the majority of genes with significant TE changes, the shifts in transcriptional and translational levels were congruent with the observed TE alteration (e.g. unchanged or decreased transcription coupled with increased translation typically elevated TE). However, a small subset of genes with significant TE alterations displayed concordant changes (Group C in both Figure 4(C and D)) at both the transcriptional and translational levels, or showed undetectable changes at these levels (Group E in both Figure 4(C and D)). This may arise from amplitude disparities and temporal asynchrony between these regulatory tiers, ultimately leading to altered gene TE, as previously observed in multi-layered regulatory networks [81,82].

Previous studies have indicated that gene sequence features influence protein abundance and TE [83,84]. We observed that genes with significantly elevated TE possess lower GC content in their CDS and higher NMFE values, indicating low stability of the mRNA secondary structure of these genes [85]. Studies indicated that this unstable mRNA secondary structure (characterized by low GC content and high NMFE) enhances mRNA translation efficiency, particularly under stress conditions [84,86,87]. These findings suggested that keratinocytes may respond to fungal infection by employing a selective translation mechanism, preferentially utilizing genes with inherently “translationally favorable” structural features (low GC, high NMFE) to facilitate a rapid response [88,89]. Such sequence characteristics may represent a molecular strategy evolved by the host to enable the swift and effective initiation of defense programs against pathogens [89]. This discovery holds potential implications for understanding cutaneous immune defense mechanisms and developing novel antifungal strategies.

Furthermore, KEGG pathway enrichment analysis of these genes revealed that those with significantly increased TE were predominantly enriched in DNA damage repair-related pathways, including: Cell cycle, Homologous recombination, p53 signaling pathway, Fanconi anemia pathway, Nucleotide excision repair, and Base excision repair [90,91]. Multiple studies demonstrate that fungal infection induces reactive oxygen species (ROS) generation and DNA double-strand breaks (DSBs) in keratinocytes, leading to oxidative damage and replication stress [33,92,93]. Our KEGG enrichment results indicated that *T. mentagrophytes* infection inflicts substantial DNA damage on these cells. In response, keratinocytes robustly activate the p53-centered DNA damage response (DDR) network [94] and rapidly mobilize DDR pathways via “translational reprogramming” to counteract this pathogen-induced genomic stress, thereby maintaining genomic stability and promoting cell survival. Conversely, genes exhibiting significantly decreased TE were significantly enriched in pathways related to inflammation and immune responses, cell growth and differentiation, and metabolism. These include the TNF signaling pathway, MAPK signaling pathway, IL-17 signaling pathway, Cytoskeleton in muscle cells, Osteoclast differentiation, Growth hormone synthesis, secretion and action, and AGE-RAGE signaling pathway in diabetic complications. Following *T. mentagrophytes* infection, keratinocytes appear to strategically divert translational resources away from energy-intensive yet non-critical functions (such as robust inflammatory responses, growth/differentiation programs, and complex stress responses) to prioritize immediate defense mechanisms and metabolic reprogramming [95]. This translational shift presents a complementary pattern to the enrichment of DNA repair pathways among TE-increased genes, collectively revealing a finely orchestrated cellular strategy under infection pressure to prioritize survival-critical responses. These findings significantly advance our understanding of the host’s translational reprogramming response to fungal challenge and provide a foundation for further mechanistic studies of fungus-host interactions.

### Beyond canonical inhibition: dual enhancement of mORF translation efficiency by uORFs and dORFs in keratinocytes

uORFs are *cis*-acting elements situated within the 5’-untranslated region (5’-UTR) that critically regulate translational initiation through modulation of ribosome scanning efficiency [96,97]. Studies have shown that uORF variations, by altering the translation of mORF, are associated with several human diseases [98,99]. For instance, a four-nucleotide deletion was identified in the uORF of CDKN1B, the gene encoding cyclin-dependent kinase inhibitor p27. This mutation extends the uORF length, leading to a significant decrease in p27 protein levels in cancer [100]. Extensive experimental data confirm that uORFs substantially attenuate the translational output of their associated mORFs in eukaryotic systems [34,35,37,56]. Contrary to their canonical inhibitory roles, functional analyses have revealed that select uORFs can actively promote the translation of downstream CDS under certain conditions [101–103]. Our results also suggested that uORFs may play a similar promoting role in the translation of the downstream coding sequence.

Additionally, we further analyzed the expression levels of genes that exhibited high transcriptional changes but no corresponding translational changes. We compared the basal expression levels (FPKM) of the upregulated/translation unchanged group and the downregulated/translation unchanged group (groups D+F in Figure 3(A)) separately against the global median of all expressed genes. The results showed that both group D and group F are significantly lower than the global median (Figure S16), indicating that low-abundance transcripts are more prone to transcriptome-translatome uncoupling. This may be explained by intrinsically lower translation efficiency of lowly expressed genes or a stronger capacity to buffer transcriptional fluctuations. Furthermore, we also analyzed the translated uORF number in the transcripts of these genes. In both group D and group F, more than 96% of the genes harbored transcripts with ≤1 translated uORF (Table S24). This indicated that, overall, these genes are not enriched for high translated uORF numbers, suggesting that alternative mechanisms – such as microRNAs, RNA-binding proteins, or regulation of translation elongation – play more dominant roles in mediating this transcriptome-translatome uncoupling. Collectively, our data demonstrated that transcriptional-translational uncoupling is enriched in low-expressing genes and is not driven by uORF abundance, providing new insights into layered control of gene expression.

Relative to the substantial body of research devoted to uORFs in 5’UTRs, dORFs in 3’UTRs have garnered comparatively less investigative focus. Studies in human, mouse, and zebrafish models have revealed that dORFs can confer a marked increase in the TE of their associated mORFs, and this effect depends on the number of dORFs rather than their length or encoded peptide products [34,35]. The regulatory functions of dORFs in modulating the TE of mORFs within keratinocytes remain systematically uncharacterized. Interestingly, our results demonstrated that both translatable uORFs and dORFs may similarly enhance the gene TE of the corresponding mORF. However, while an increased number of translatable uORFs does not amplify the TE enhancement, a higher number of translatable dORFs results in a stronger TE enhancement. This latter observation regarding dORFs aligns specifically with the findings of Wu et al. [34]. Significantly, sequence features differ significantly between untranslatable and translatable ORFs (uORFs and dORFs), suggesting these features may be closely linked to ORF expression. Together, these findings provide new insights into the effects of both uORFs and dORFs on the TE of mORFs in keratinocytes.

### Regulation of translation efficiency of mORF by miRNAs, uORFs, and dORFs in keratinocytes

miRNAs constitute an abundant class of small non-coding RNAs generated through the stepwise cleavage of hairpin-containing precursor transcripts, and hundreds of distinct human miRNAs have been cataloged to date [104,105]. In addition to being regulated by their own uORFs and dORFs, gene expression and translation are also modulated by miRNAs [106,107]. Consistent with numerous existing studies [108–110], our analysis revealed that miRNA suppresses both the transcription and translation of corresponding target genes in keratinocytes, while also reducing the TE of these genes. Within the broader context of gene regulation involving both uORFs and miRNAs, research in Drosophila demonstrated that uORFs primarily drive the suppressive effect on TE, while miRNAs play a secondary, auxiliary role [111]. However, our findings revealed a distinct regulatory dynamic in keratinocytes under normal physiological conditions: uORFs significantly promote the TE of mORF genes, whereas miRNAs function to suppress TE. Collectively, our results suggested that uORFs exert a more dominant positive influence on mORF TE than the inhibitory effect exerted by miRNAs under normal physiological conditions. Regarding dORFs and miRNAs, it was shown that dORFs are key regulators of TE [44]. However, our data remain inconclusive as to which exerts a predominant regulatory influence on TE under the experimental conditions examined. Future studies should therefore clarify the complex interplay between these regulators in stressed keratinocytes. Based on the finding that uORFs enhance TE, we speculate that this effect may involve mechanisms such as regulated reinitiation, leaky scanning, and translational damper/buffer, among others [112,113]. In addition, we found that uORFs may attenuate miRNA-mediated suppression. One plausible explanation is that uORF translation promotes mORF translation through a reinitiation mechanism, thereby indirectly enhancing mRNA stability and counteracting miRNA-mediated degradation, as efficiently translated mRNAs are generally more resistant to decay machinery [114]. Alternatively, although steric occlusion of miRNA-RISC complexes by the translational machinery is thought to operate primarily within the coding sequence and the proximal 3′ UTR, long-range mRNP conformational changes induced by ribosome occupancy on the uORF could potentially alter 3′ UTR target site accessibility. However, these hypotheses remain speculative, and further studies are required to clarify the specific mechanisms underlying both the TE enhancement and the attenuation of miRNA suppression.

In summary, by integrating Ribo-seq and RNA-seq, we demonstrated the pivotal role of translational regulation in the keratinocyte response to *T. mentagrophytes* infection. These findings provide the first genome-wide map of translatable ORFs and translational dynamics in dermatophyte-infected keratinocytes, uncovering how sequence features (e.g. GC content, NMFE), *cis*-regulatory elements (uORFs/dORFs), and miRNAs orchestrate TE. Collectively, we elucidate a multi-tiered translational control strategy underlying epidermal antifungal immunity, offering a molecular framework for targeting host-pathogen interactions in dermatophytosis.

### Limitations and future perspectives

This study has several technical limitations. Firstly, we did not validate our findings in an in vivo or organotypic skin model. This highlights the inherent physiological limitations of the in vitro model: the experiments were based on a keratinocyte infection model in vitro, which fails to replicate the complex microenvironment in vivo, the paracrine effects of immune cells, and the physical barrier function of the stratum corneum. Consequently, the physiological relevance of the observed host-pathogen interactions may be limited. Future research should validate the core findings in skin organoids [115,116] or humanized mouse models [117,118] to accurately assess the contribution of uORFs, dORFs, and the miRNA network within an in vivo context and evaluate their potential as therapeutic targets against fungal pathogens [119]. To bridge this gap in a more physiologically relevant but controlled setting, we are currently establishing a skin organoids model to validate a subset of our key findings.

Secondly, the mechanistic insights remain incomplete. Although this study revealed that uORFs/dORFs and miRNAs collaboratively regulate TE, the molecular details underlying how uORFs and dORFs enhance the translation of the mORF – such as ribosome reinitiation, alterations in ribosomal scanning modes, or structural unwinding dynamics – remain unelucidated [120–122]. Similarly, whether miRNAs functionally compete with or synergize with uORFs/dORFs requires further elucidation through experimental approaches.

Third, functional annotation and evolutionary characterization of lncORFs remain inadequate. While our bioinformatic analysis identified over 185,000 LncORFs, their coding potential and biological significance are still confined to sequence characteristics and expression correlations. In particular, we were unable to perform systematic analyses of evolutionary conservation or putative functional domains for these lncORFs, owing to the lack of well-annotated reference functional genes and the generally poor cross-species conservation of lncORFs. Future work should experimentally verify their translated products through mass spectrometry-based peptidomics [123] and antibody labeling, coupled with in vitro and in vivo phenotypic rescue/knockout assays using gene editing (CRISPR-Cas9 or CRISPRi) [124,125]. This integrated approach will enable systematic screening of differentially expressed LncORFs for functional micropeptide candidates and elucidate their effector mechanisms in antifungal immunity [126]—such as modulation of inflammatory pathways or barrier repair processes.

Fourth, a key limitation is that we did not perform CRISPR validation of uORF/dORF functions. Although correlative evidence from *BMP4* uORFs suggests regulatory roles, the involvement of these uORFs in antifungal defense has not been causally demonstrated. Future experiments deleting the uORF of *BMP4* will be required to test the causal effects of these deletions on fungal burden and cytokine secretion.

Finally, a limitation of this study is the lack of direct functional assays (e.g. fungal growth inhibition, barrier permeability) linking specific TE changes to infection outcome. While we observed that genes with elevated TE are enriched in DDR network pathways – providing candidate pathways for targeted intervention – functional validation remains pending. Specifically, it remains untested whether pharmacological or oligonucleotide-based modulation of TE can enhance the antifungal resilience of keratinocytes. Future studies should prioritize both direct phenotypic assays and the development of small-molecule modulators targeting hub genes with significant TE alterations (e.g. p53 signaling nodes in DDR pathways or key regulators of IL-17/TNF signaling) to evaluate their therapeutic potential for augmenting host defense mechanisms.

## Conclusions

This study establishes the first multi-omics (Ribo-seq/RNA-seq) atlas of *T. mentagrophytes*-induced translational reprogramming in human keratinocytes, revealing: (1) Global transcriptome-translatome discordance with TE remodeling redirecting resources from inflammation to DNA repair. (2) Non-canonical ORF networks where uORFs/dORFs enhance mORF TE and buffer miRNA suppression. (3) RNA structural stability (high GC/low NMFE) as a key coordinator of multi-layer regulation. These findings decode host translational adaptation to dermatophytes and nominate TE-centric targets for antifungal strategies.

## Supplementary Material

Supplemental Material

Figure S7.tif

Figure S14.tif

Figure S15.tiff

Figure S12.tif

Figure S6.tif

Figure S4.tif

Figure S10.tif

Figure S13.tif

Figure S5.tif

Figure S8.tif

Figure S2.tif

Figure S16.tif

Figure S11.tif

Figure S9.tif

Supplementary material legends.docx

Figure S3.tif

Figure S1.tiff

## Data Availability

The raw sequence data (Ribo-seq and RNA-seq) that support the findings of this study are openly available in the Genome Sequence Archive [127] at National Genomics Data Center [128] at https://ngdc.cncb.ac.cn/gsa-human/browse/HRA012603, reference number HRA012603. The raw sequence data from a previous miRNA study are also openly available in the NCBI Sequence Read Archive (SRA) at https://www.ncbi.nlm.nih.gov/sra/?term= under accession numbers SRX22121525–SRX22121530. The datasets generated and analyzed during this study, along with all supplementary figures and tables, are openly available in the figshare repository at https://doi.org/10.6084/m9.figshare.30556448 [129].

## Abbreviations

CDS: Coding Sequence
circRNAs: Circular RNAs
DDR DNA: Damage Response
dORFs: Downstream Open Reading Frames
DSBs DNA: Double-strand Breaks
FPKM: Fragments Per Kilobase of transcript per Million mapped reads
GO: Gene Ontology
KEGG: Kyoto Encyclopedia of Genes and Genomes
lncORFs: Long Non-coding RNA-derived ORFs
lncRNAs: Long Non-Coding RNAs
log2FC: Log2 Fold Change
miRNAs: MicroRNA
mORFs: Major Open Reading Frames
mRNA: Messenger RNA
NMFE: Normalized Minimal Free Energy
Nt: Nucleotides
ORFs: Open Reading Frames
RFs: Ribosome-protected fragments
Ribo-seq: Ribosome Profiling
RNA-seq: RNA sequencing
ROS: Reactive Oxygen Species
RPF: Ribosome-Protected mRNA Fragments
rRN: Ribosomal RNA
TE: Translational Efficiency
*T. mentagrophytes*: *Trichophyton mentagrophytes*
tRNA: Transfer RNA
uORFs: Upstream open reading frames
3’UTRs: 3’Untranslated Regions
5’UTRs: 5’Untranslated Regions
