# Supplementary figures and images for: Global translational reprogramming in Trichophyton mentagrophytes-infected keratinocytes
Source: Virulence. 2026 Aug 2;17(1):2710548. doi: 10.1080/21505594.2026.2710548 (PMC13432870; doi:10.1080/21505594.2026.2710548)

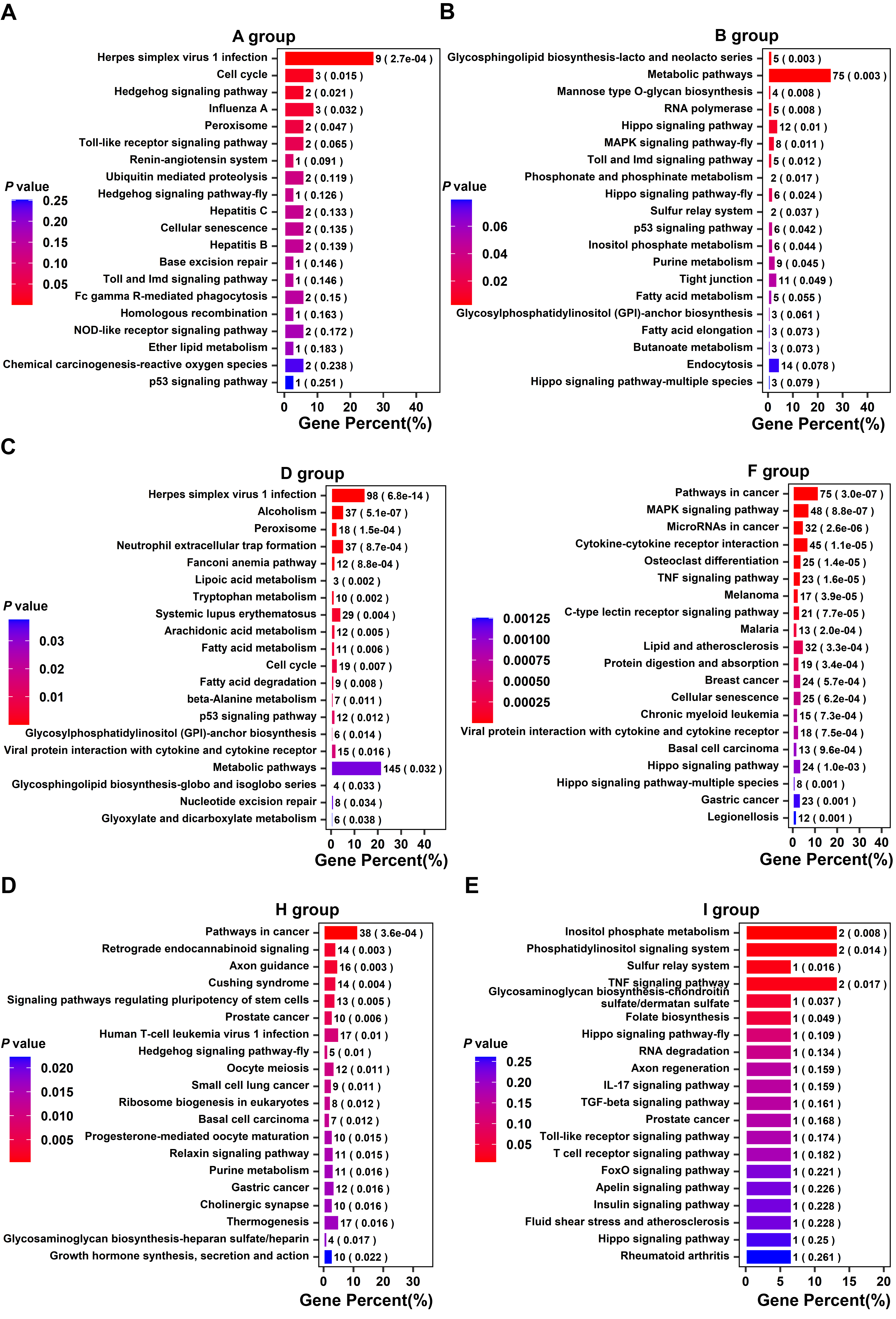

Supplement: Figure S7.tif [file KVIR_A_2710548_SM8753.tif]

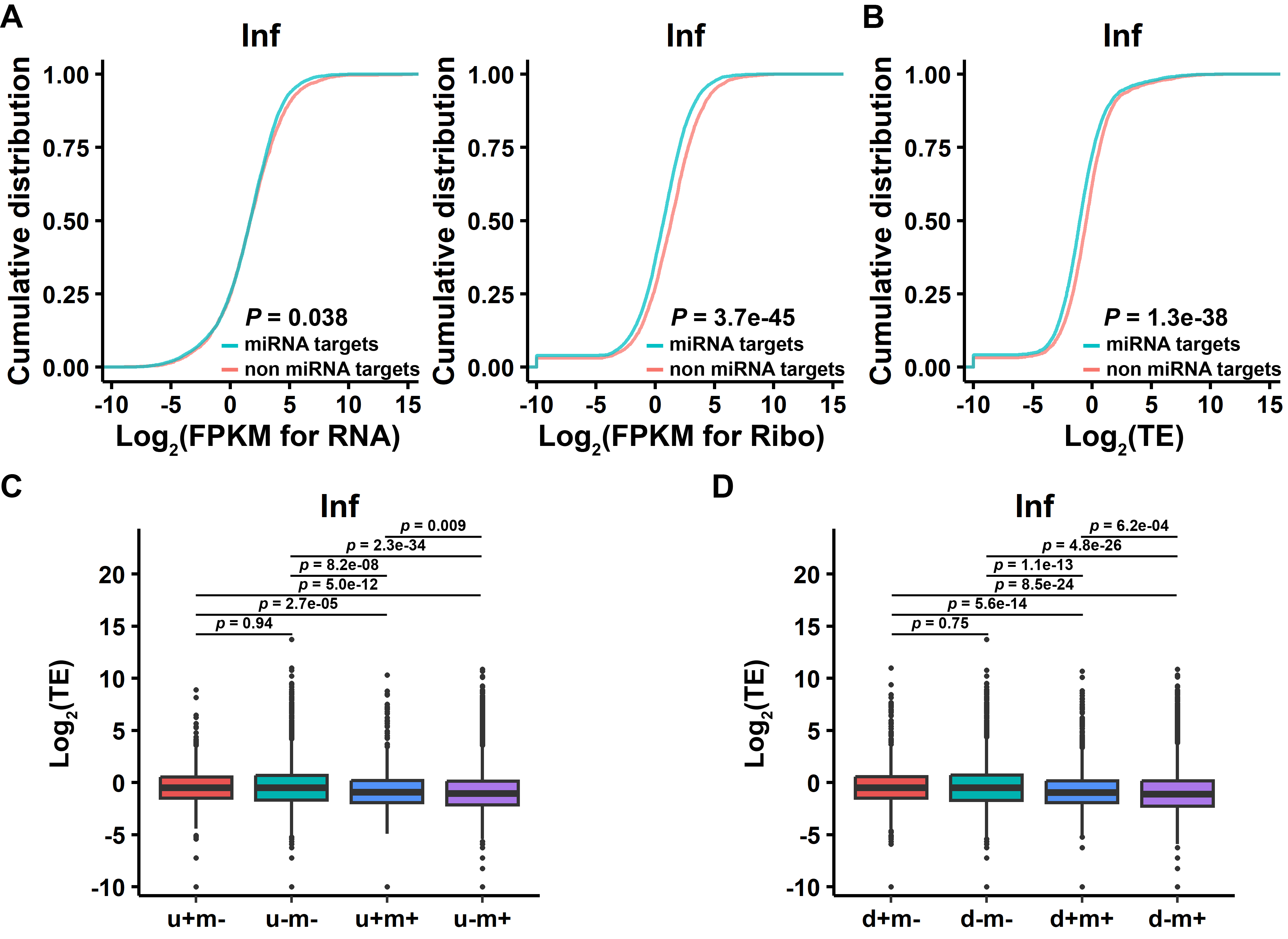

Supplement: Figure S14.tif [file KVIR_A_2710548_SM8752.tif]

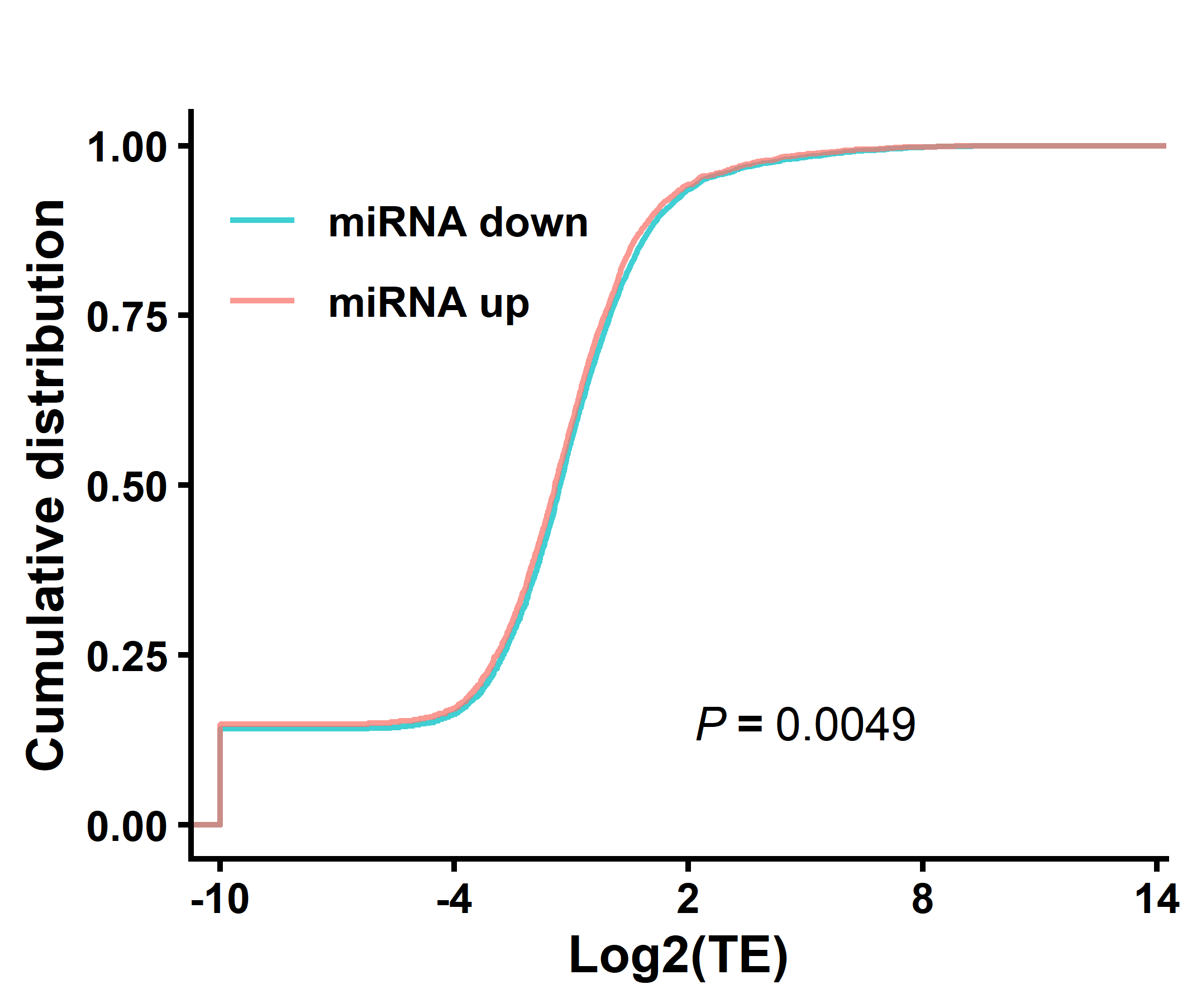

Supplement: Figure S15.tiff [file KVIR_A_2710548_SM8751.tiff]

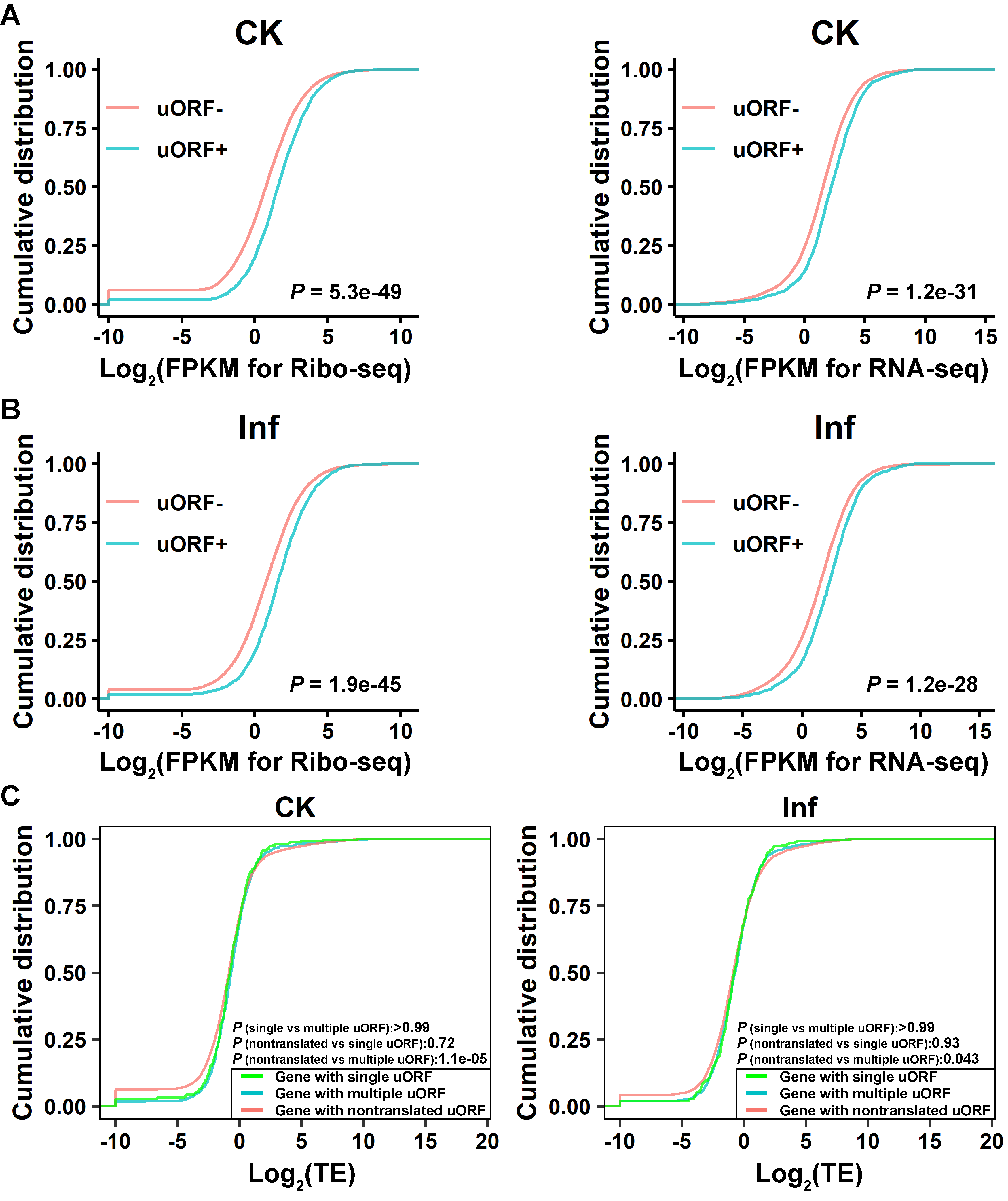

Supplement: Figure S12.tif [file KVIR_A_2710548_SM8750.tif]

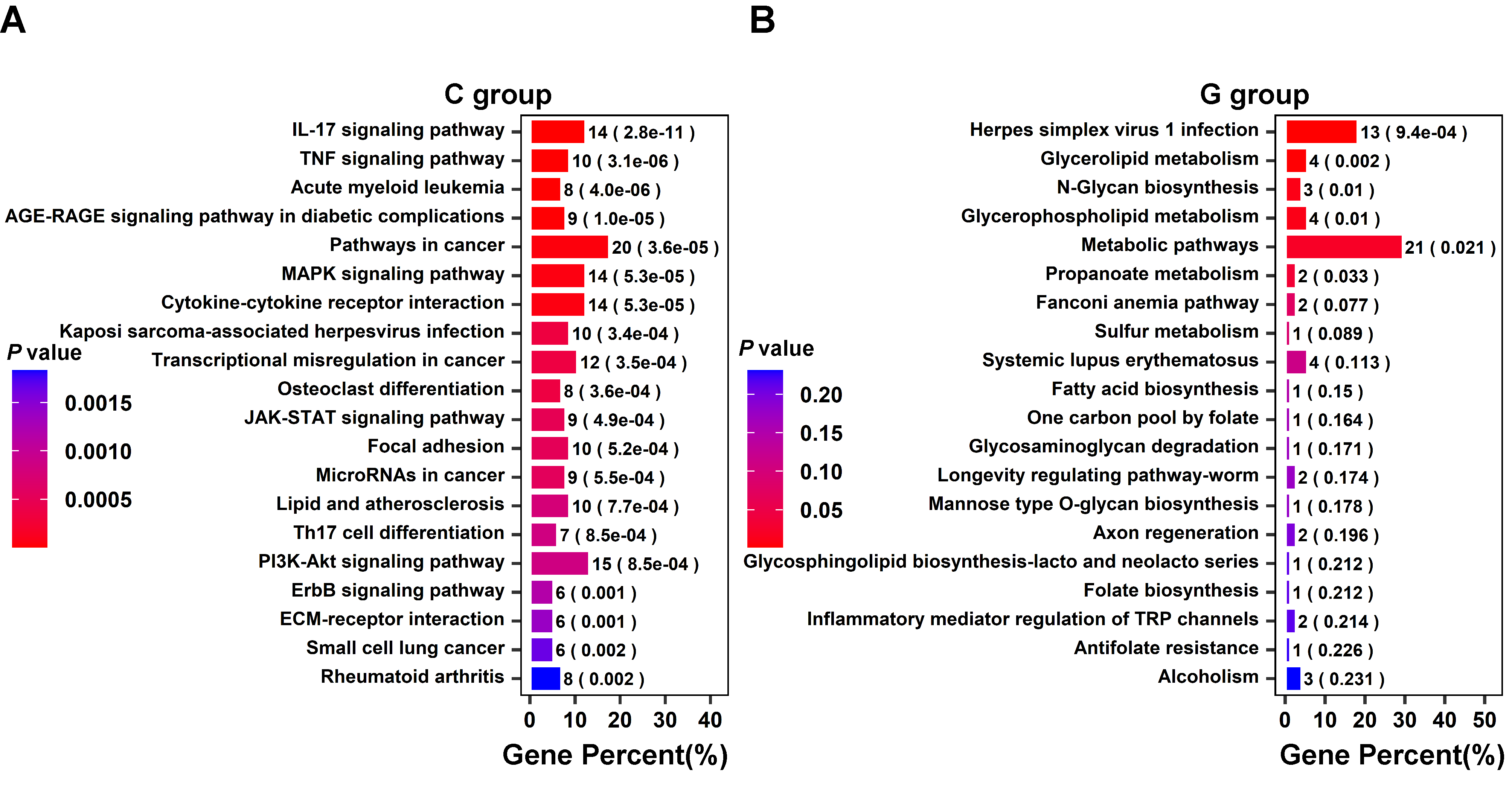

Supplement: Figure S6.tif [file KVIR_A_2710548_SM8749.tif]

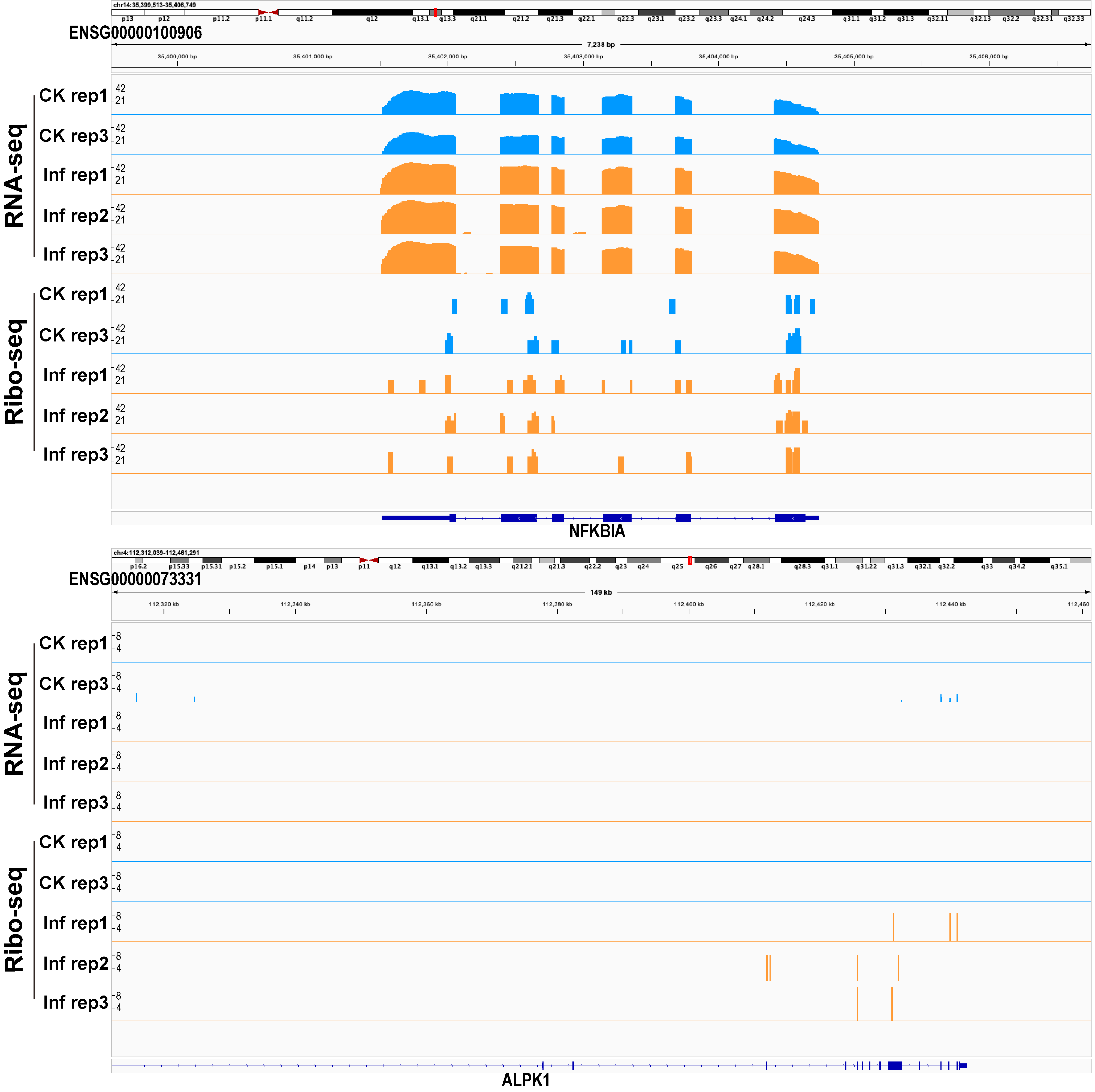

Supplement: Figure S4.tif [file KVIR_A_2710548_SM8748.tif]

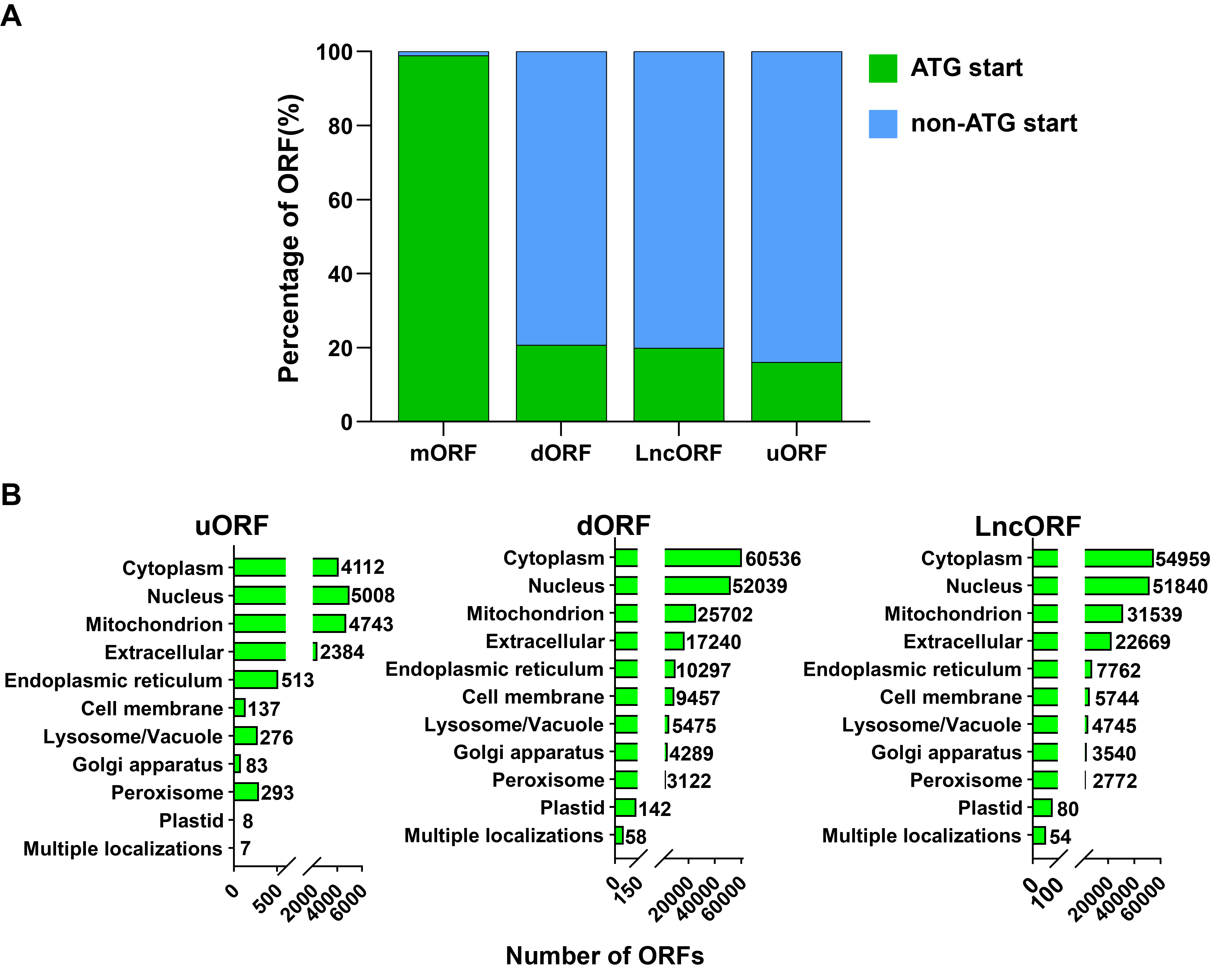

Supplement: Figure S10.tif [file KVIR_A_2710548_SM8747.tif]

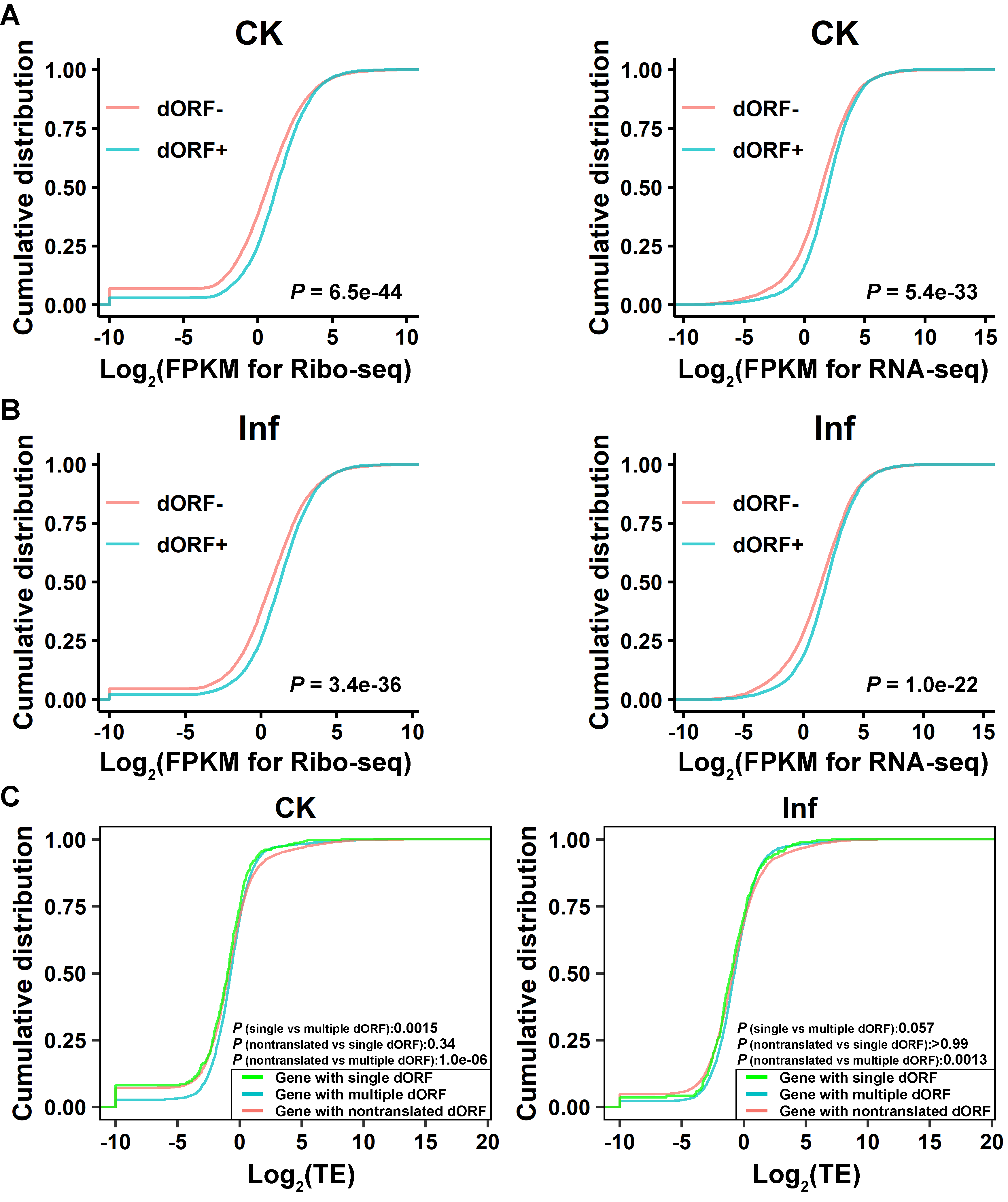

Supplement: Figure S13.tif [file KVIR_A_2710548_SM8746.tif]

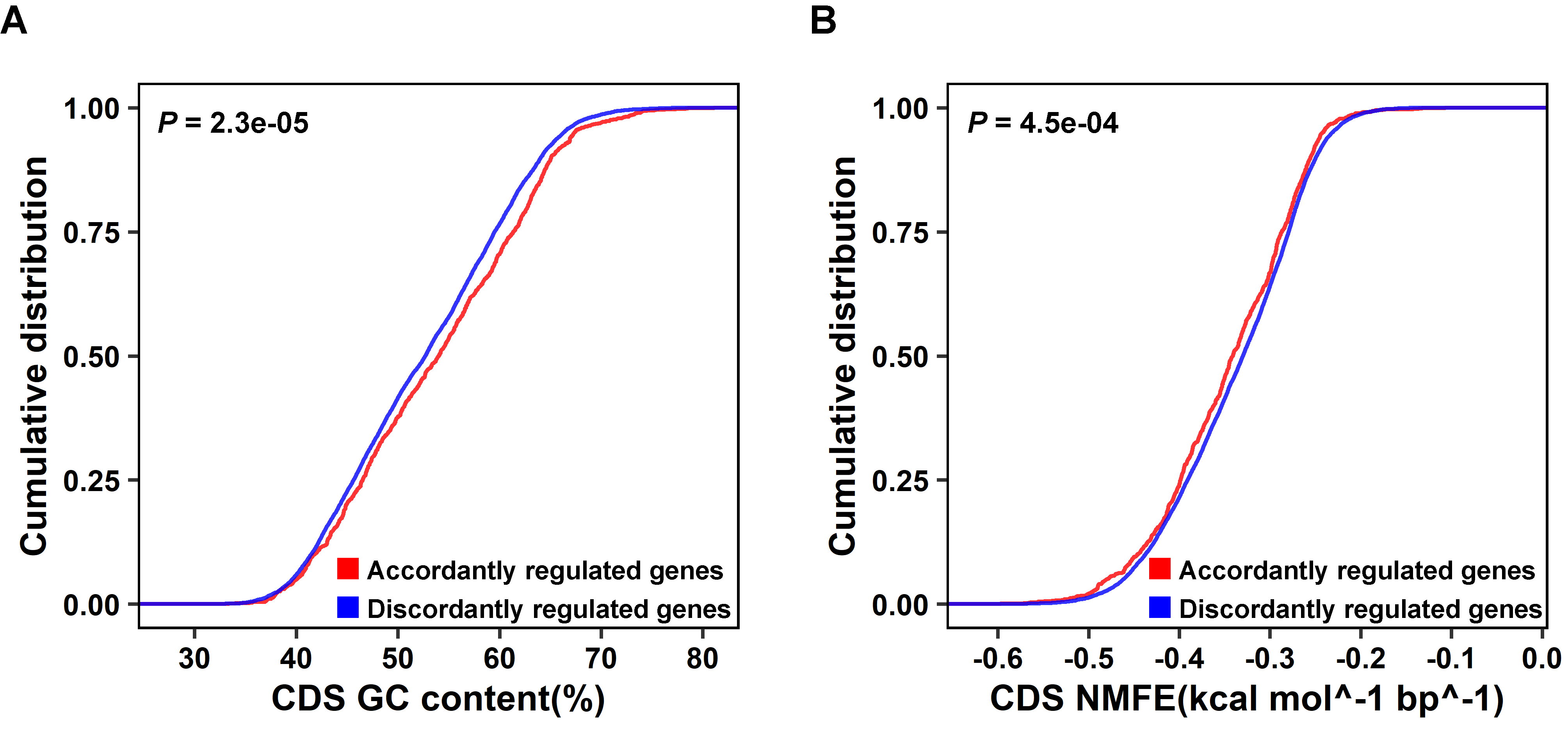

Supplement: Figure S5.tif [file KVIR_A_2710548_SM8745.tif]

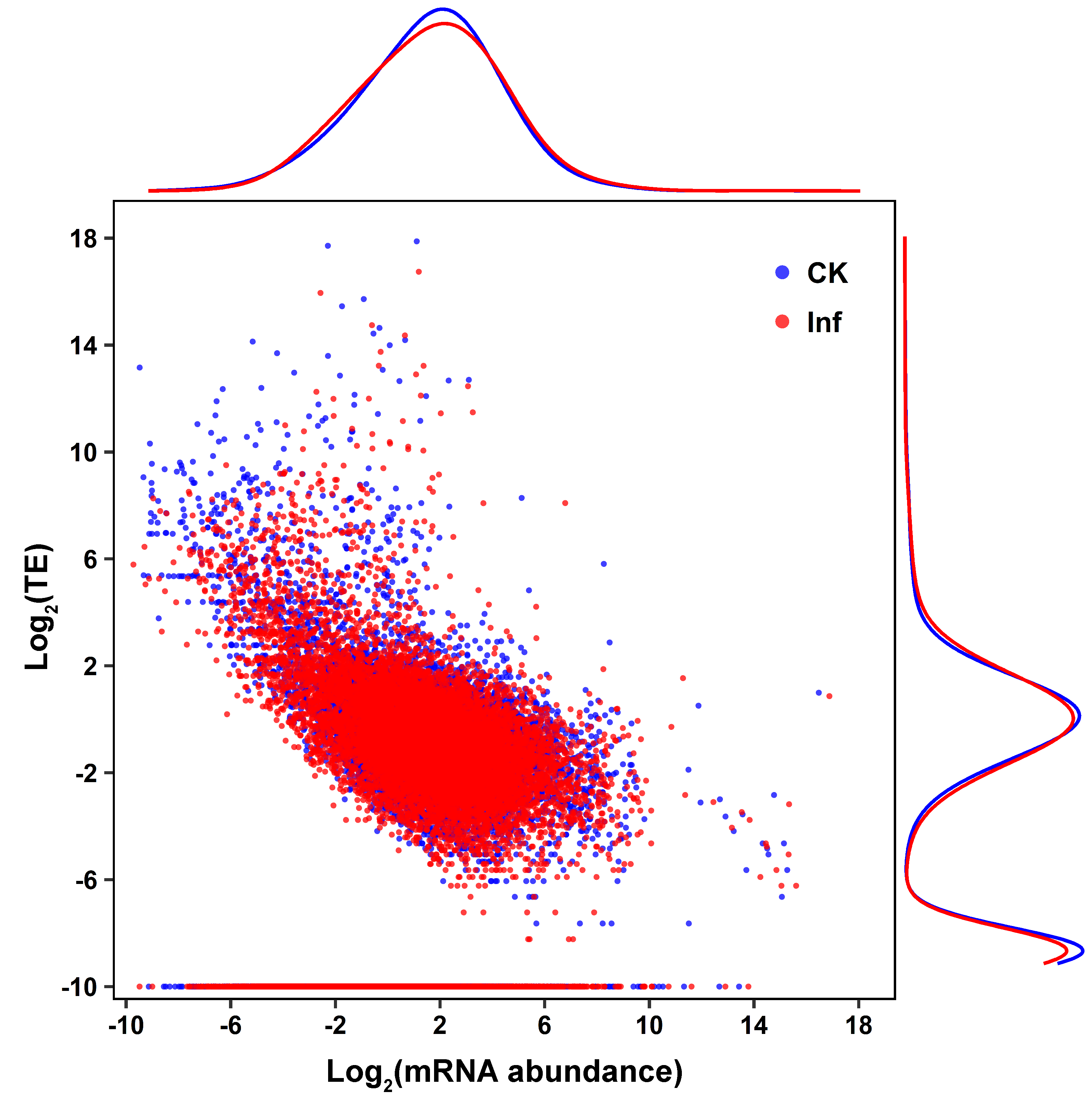

Supplement: Figure S8.tif [file KVIR_A_2710548_SM8744.tif]

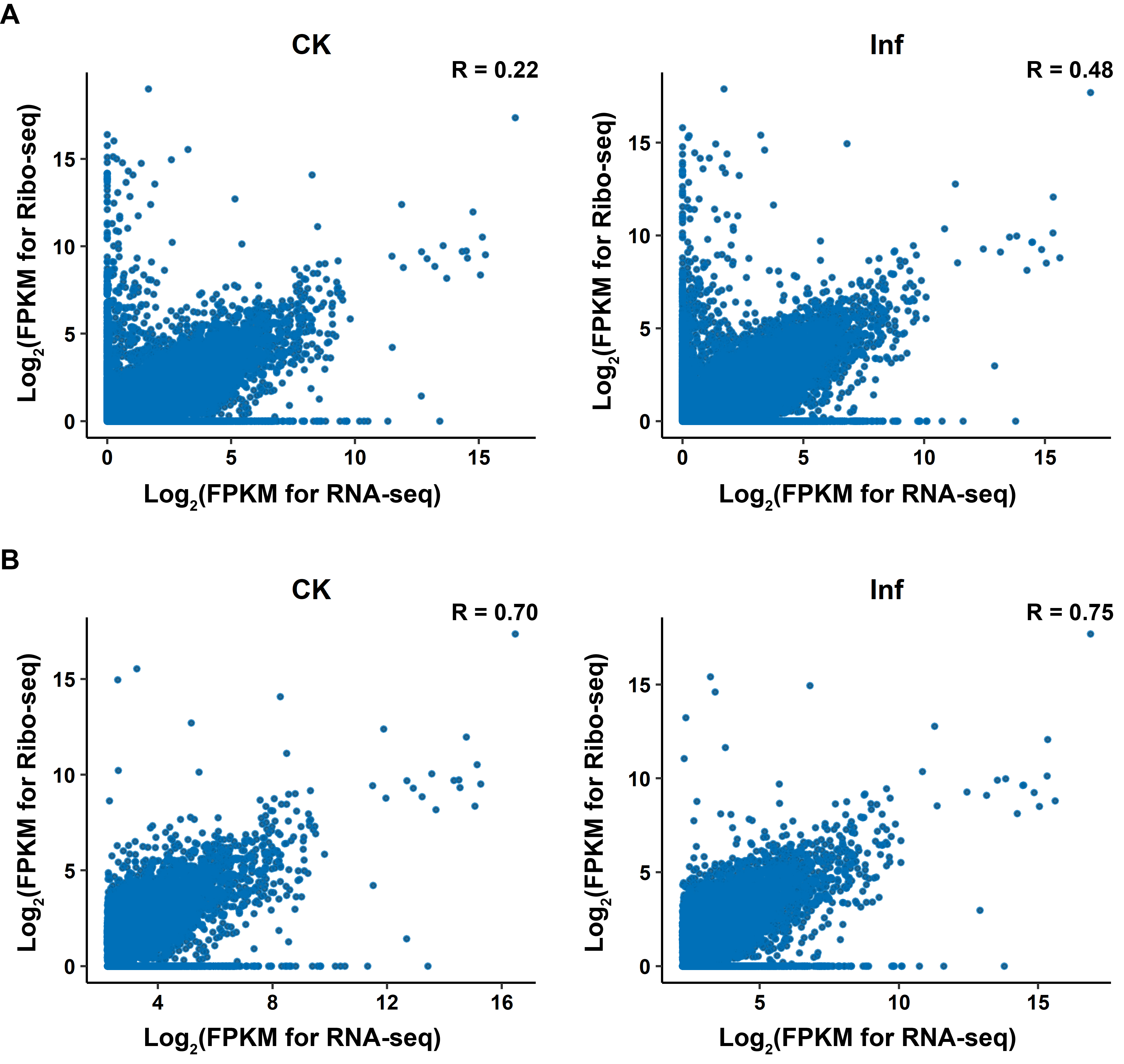

Supplement: Figure S2.tif [file KVIR_A_2710548_SM8743.tif]

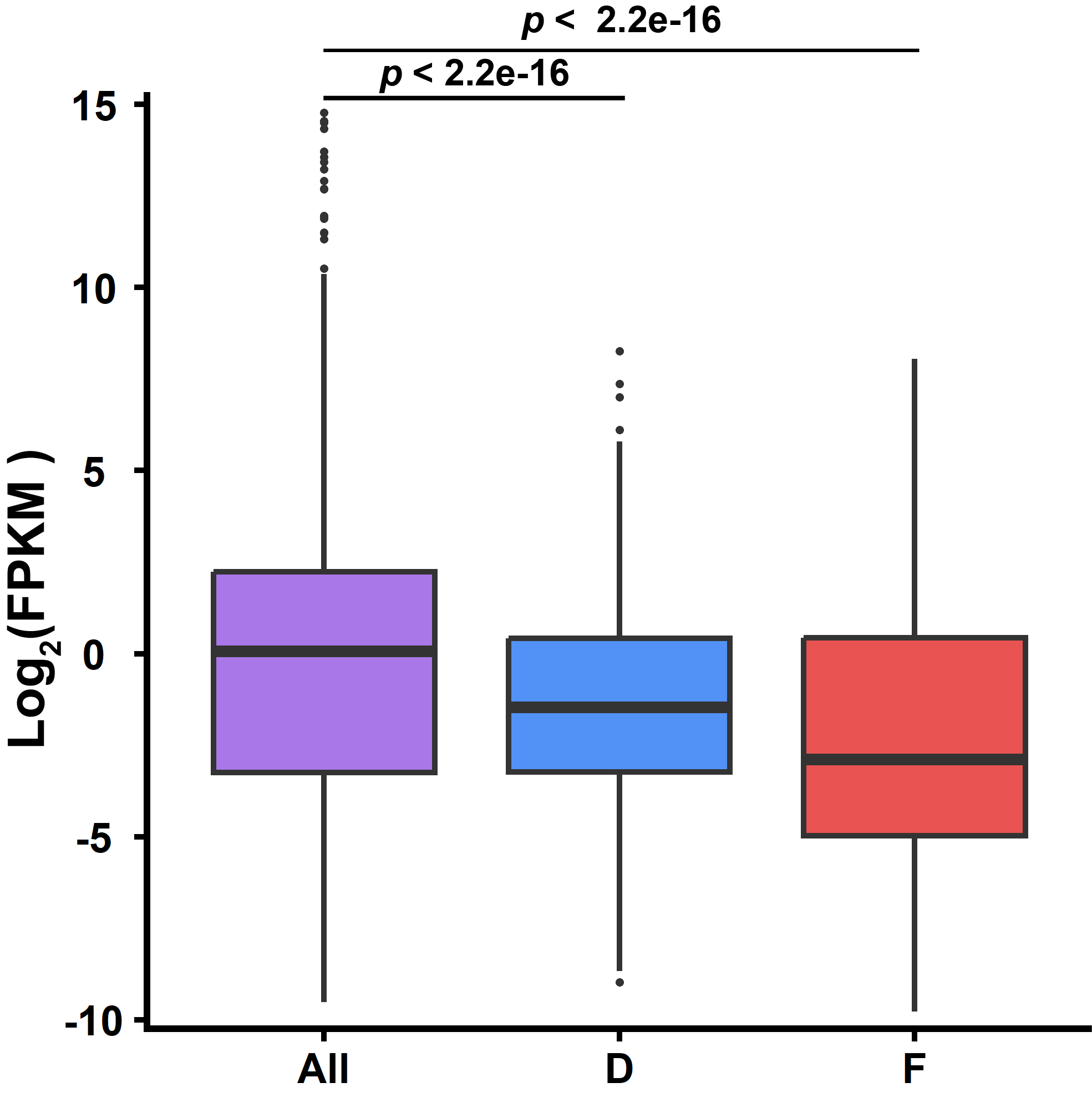

Supplement: Figure S16.tif [file KVIR_A_2710548_SM8742.tif]

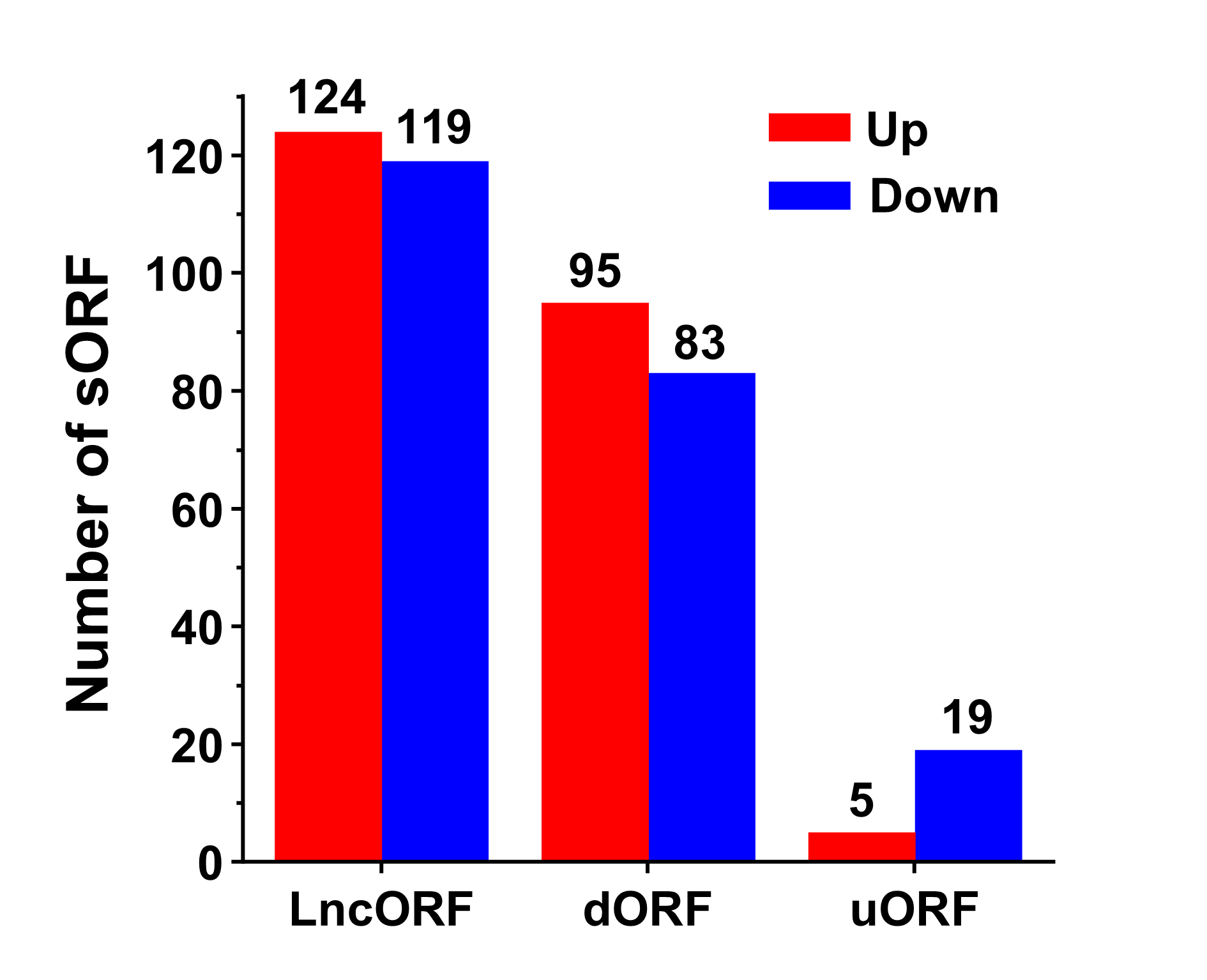

Supplement: Figure S11.tif [file KVIR_A_2710548_SM8741.tif]

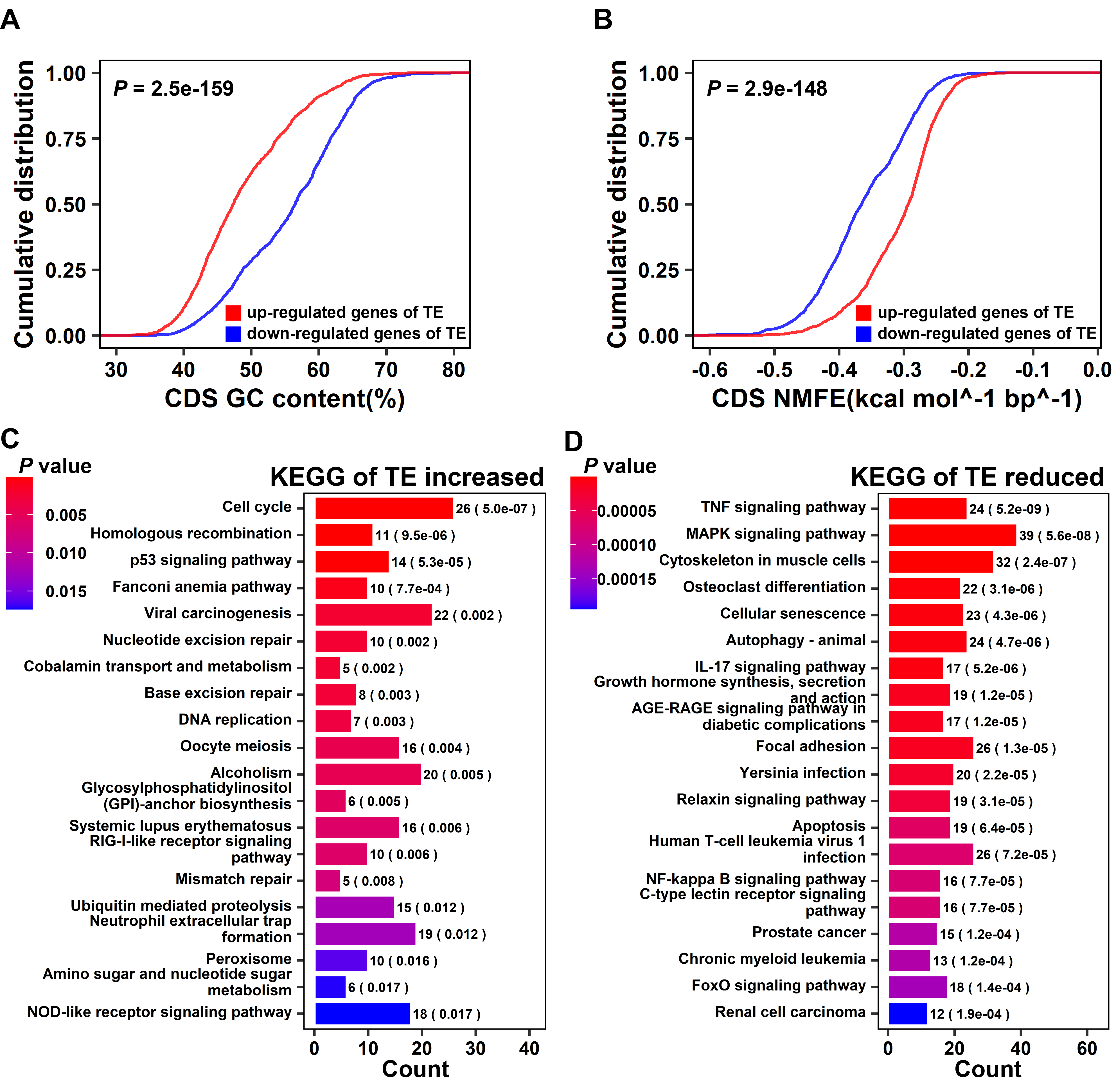

Supplement: Figure S9.tif [file KVIR_A_2710548_SM8740.tif]

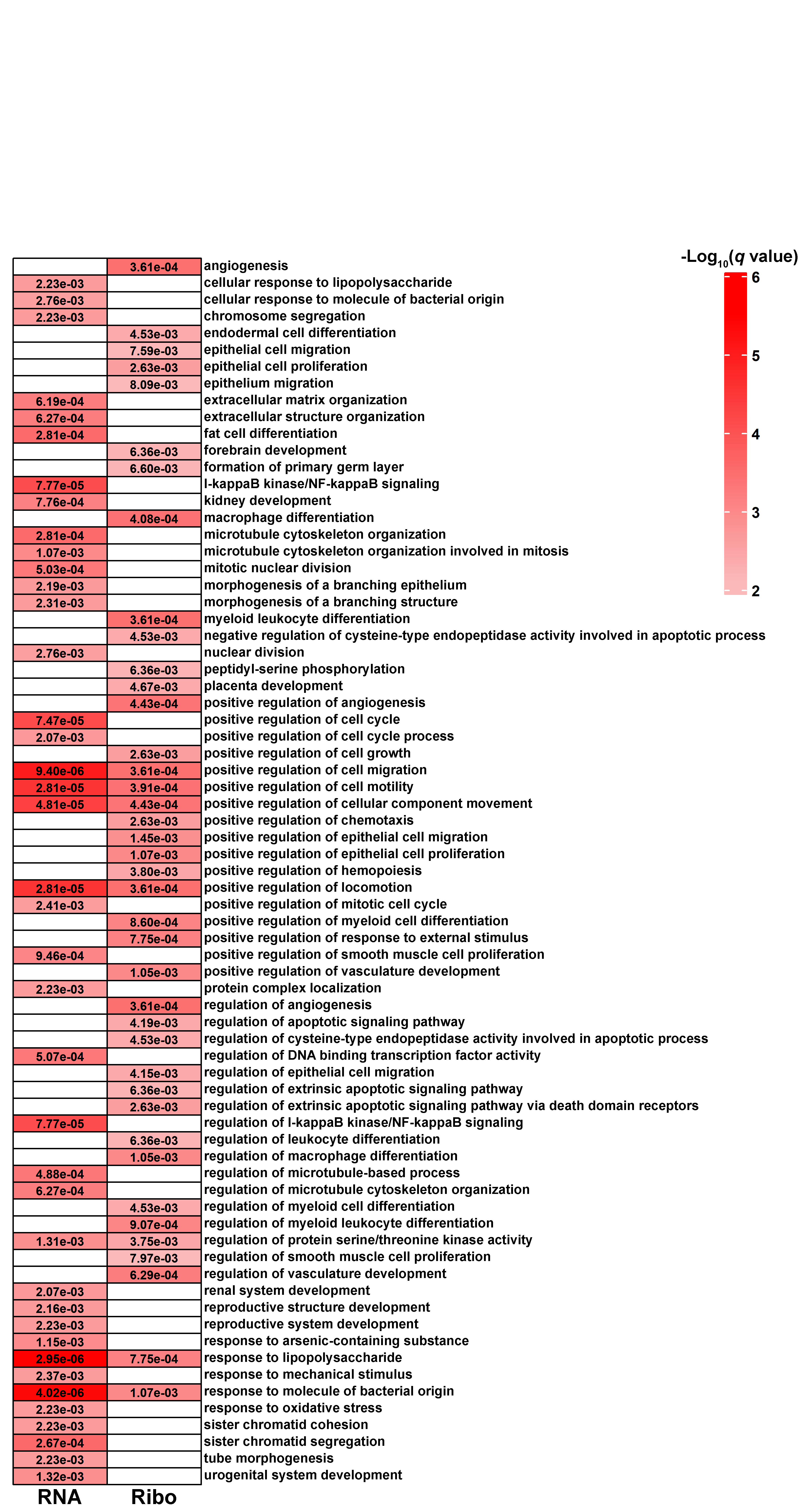

Supplement: Figure S3.tif [file KVIR_A_2710548_SM8738.tif]

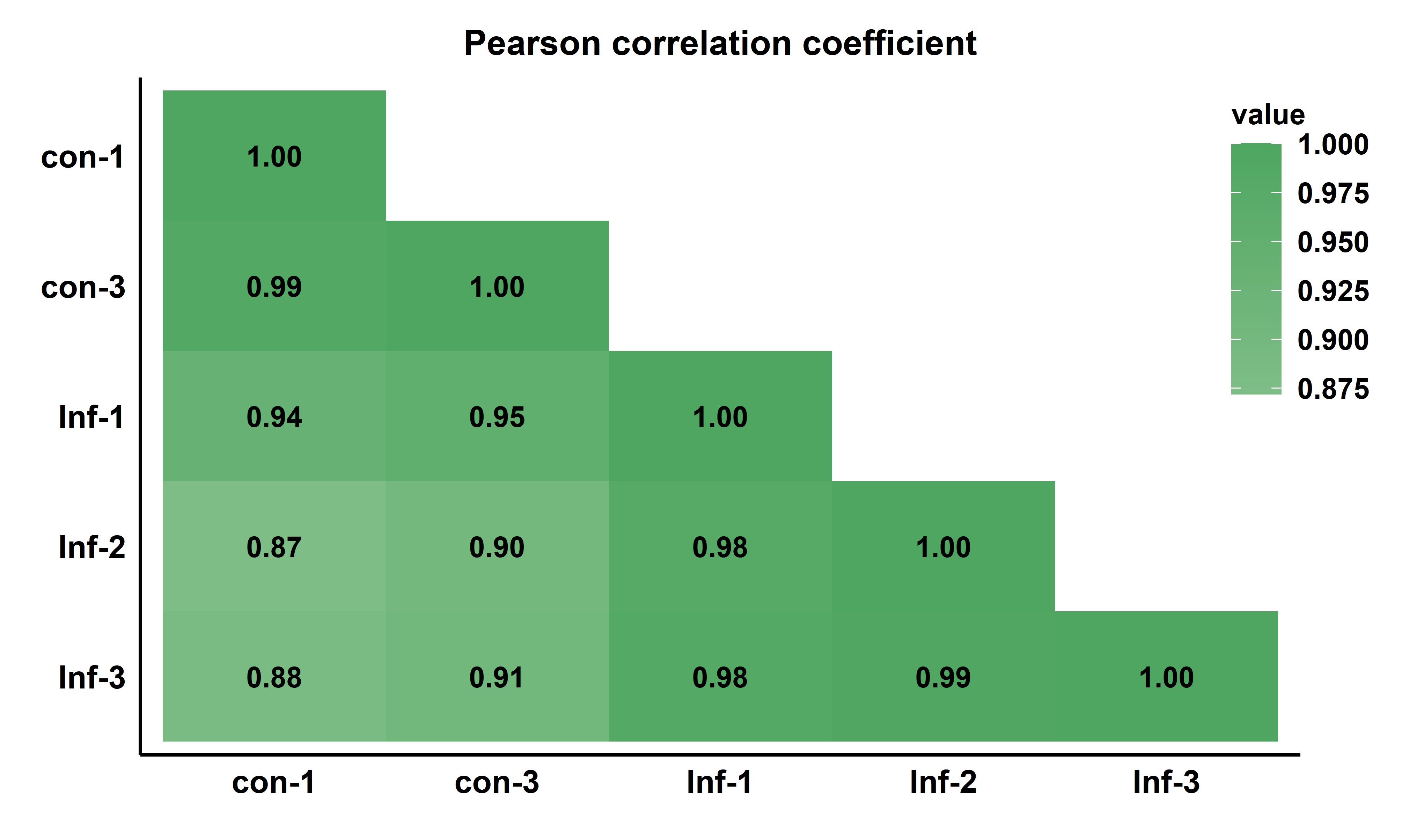

Supplement: Figure S1.tiff [file KVIR_A_2710548_SM8737.tiff]
