# Supplementary material for: Global translational reprogramming in Trichophyton mentagrophytes-infected keratinocytes
Source: Virulence. 2026 Aug 2;17(1):2710548. doi: 10.1080/21505594.2026.2710548 (PMC13432870; doi:10.1080/21505594.2026.2710548)
Supplement: Supplementary material legends.docx [file KVIR_A_2710548_SM8739.docx]

**supplementary material (figure and tables) legends**

**Figure S1**. Correlation efficiency among the five samples based on ribosome profiling data.

**Figure S2.** The correlation of FPKM results between RNA-seq and Ribo-seq in the CK and Inf groups. (A) All transcripts. (B) Highly expressed transcripts (FPKM > 0 and FPKM ≥ the 75th percentile).

**Figure S3.** Enrichment of biological processes associated with *T. mentagrophytes* infection at transcriptional and translational levels (top 40 entries per level).

**Figure S4.** IGV browser view for two genes, ENSG00000100906 and ENSG00000073331.

**Figure S5.** Comparison of GC contents and NMFE between concordantly regulated genes and discordantly regulated genes. (A) GC contents; (B) NMFE.

**Figure S6.** KEGG analysis of groups C and G in concordant genes. (A) groups C; (B) groups G.

**Figure S7.** KEGG analysis of groups A, D, B, F, H and I in discordant genes. (A) groups A; (B) groups B; (C) groups D and F; (D) groups H; (E) groups I.

**Figure S8.** The distribution of gene expression at the transcriptional level and TEs in the CK and Inf groups.

**Figure S9.** Analysis of genes with significantly increased and reduced of TE. (A) Comparison of GC contents of genes with significantly increased and reduced of TE. (B) Comparison of NMFE of genes with significantly increased and reduced of TE. (C) KEGG analysis of genes with significantly increased TE. (D) KEGG analysis of genes with significantly reduced TE.

**Figure S10.** ORF (uORFs, dORFs and LncORFs) analysis. (A) Start-codon usage of the identified ORFs. (B) Predicted subcellular localization of these ORFs.

**Figure S11.** Differentially expressed uORFs, dORFs and lncORFs under CK and *T. mentagrophytes* infection

**Figure S12.** Comparison of gene expression and TE in mRNAs with translated and untranslated uORFs. (A, B) Comparison of transcriptional and translational gene expression levels between mRNAs with translated uORFs and mRNAs with untranslated uORFs; A, under normal conditions; B, under *T. mentagrophytes* infection conditions. (C) The distribution of TEs for mRNAs with nontranslated, single, multiple uORFs under CK and Inf.

**Figure S13.** Comparison of gene expression and TE in mRNAs with translated and untranslated dORFs. (A, B) Comparison of transcriptional and translational gene expression levels between mRNAs with translated dORFs and mRNAs with untranslated dORFs; A, under normal conditions; B, under *T. mentagrophytes* infection conditions. (C) The distribution of TEs for mRNAs with nontranslated, single, multiple dORFs under CK and Inf.

**Figure S14.** Regulation of gene expression and TEs by miRNAs, uORFs, and dORFs under *T. mentagrophytes* infection conditions. (A) Distribution of gene expression at the transcriptional level for miRNA targets and non-miRNA targets. (B) Distribution of TEs for miRNA targets and non-miRNA targets. (C) Comparison of TEs across four mRNA categories: "u+m-" (n = 587), "u-m−" (n = 3065), "u+m+" (n = 976), and "u-m+" (n = 6072). (D) Comparison of TEs across four mRNA categories: "d+m-" (n = 1365), "d-m-" (n = 2287), "d+m+" (n = 2560), and "d-m+" (n = 4488). *P* values were determined by a two-sided Wilcoxon test.

**Figure S15.** Distribution of TEs among target genes of differentially expressed miRNAs upon *T. mentagrophytes* infection.

**Figure S16.** Comparison of basal transcript abundance (FPKM) of group D and group F genes (from Figure 3A) with the global background. Genes with zero FPKM in the control condition were excluded from all analyses. The global baseline (“All genes”) was then defined as all remaining expressed genes excluding those assigned to group D or group F.

**Table Legends**

**Table S1.** Alignment statistics of for ribosome profiling reads.

**Table S2.** RNA-seq and Ribo-seq Expression Levels across the Five Samples.

**Table S3.** Analysis of Ribosome Fragment Length Distribution and Regional Mapping.

**Table S4.** Differentially expressed genes at the transcriptional and translational levels under *T. mentagrophytes* infection.

**Table S5.** GO analysis of the differentially expressed genes.

**Table S6.** List of nine different category of responsive genes under *T. mentagrophytes* infection.

**Table S7.** Important genes related to *T. mentagrophytes* infection.

**Table S8.** Sequence features between concordant and discordant genes.

**Table S9.** GO analysis of the nine different types of responsive genes.

**Table S10.** Gene Expression at Transcriptional Level and Corresponding Translation Efficiency.

**Table S11.** Genes with significant changes in gene translation efficiency.

**Table S12.** Sequence features of Genes with significantly altered of TE.

**Table S13.** uORF information.

**Table S14.** dORF information.

**Table S15.** LncORF information.

**Table S16.** Length distribution and Start-codon usage of ORFs.

**Table S17.** Subcellular Localization of uORFs, dORFs, and LncORFs.

**Table S18.** Characteristics of Translated ORFs and Untranslated ORFs.

**Table S19.** Expression Profiling of Start Codon Usage Types.

**Table S20.** Differentially expressed ORFs between the CK and Inf groups.

**Table S21.** Comparison of Gene Expression and TE Regulated by uORFs, dORFs, and miRNAs.

**Table S22.** Identified miRNA target genes.

**Table S23.** Differentially expressed miRNAs.

**Table S24.** Number of translated uORFs for genes in Groups D and F.
